# Direct Three-Dimensional Mass Spectrometry Imaging with Laser Ablation Remote Atmospheric Pressure Photoionization/Chemical Ionization

**DOI:** 10.1021/acs.analchem.4c03402

**Published:** 2024-07-30

**Authors:** Tomasz Ruman, Zuzanna Krupa, Joanna Nizioł

**Affiliations:** † Department of Inorganic and Analytical Chemistry, Faculty of Chemistry, Rzeszów University of Technology, 6 Powstan ´ców Warszawy Ave., Rzeszów 35-959. Poland; ‡ Doctoral School of Engineering and Technical Sciences at the Rzeszów University of Technology, 8 Powstan ´ców Warszawy Ave., Rzeszów 35-959, Poland

## Abstract

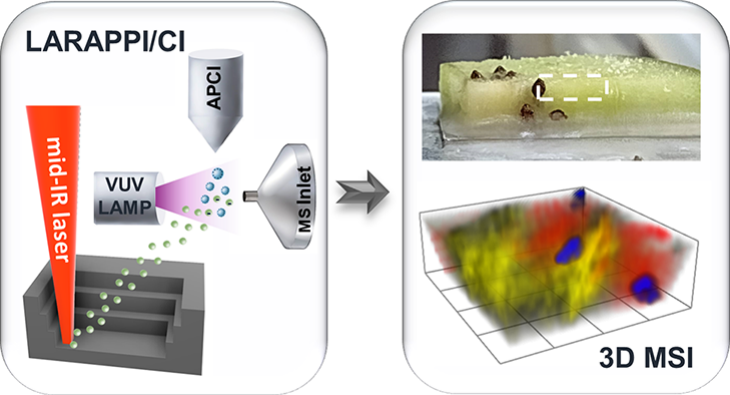

The laser ablation remote atmospheric pressure photoionization/chemical ionization (LARAPPI/CI) platform coupled to an ultrahigh resolution quadrupole-time-of-flight (QToF) mass spectrometer was developed and employed for the first direct three-dimensional (3D) mass spectrometry imaging (MSI) of metabolites in human and plant tissues. Our solution for 3D MSI does not require sample modification or cutting into thin slices. Ablation characteristics of an optical system based on a diffraction optical element are studied and used for voxel stacking to directly remove layers of tissues. Agar gel, red radish, kiwi, human kidney cancer, and normal tissue samples were used for the tests of this new system. The 2D and 3D ion images vividly illustrate differences in the abundances of selected metabolites between cancerous and noncancerous regions of the kidney tissue and also between different parts of plant tissues. The LARAPPI/CI MSI setup is also the first example of the successful use of combined dopant-assisted atmospheric pressure photoionization (DA-APPI) and atmospheric pressure chemical ionization (APCI) ion source for mass spectrometry imaging.

## Introduction

Mass spectrometry imaging (MSI) has emerged as an essential technology that offers the simultaneous analysis of a broad spectrum of molecular species with unparalleled chemical specificity.^1^ This technique excels in its ability to identify a diverse array of molecules, including endogenous and exogenous compounds, without the need for labels in a single experiment on the same tissue section.^2^ It enables the detailed mapping of molecular distributions, the identification of post-translational modifications, and the acquisition of relative quantitative data across various samples.^3^ With its high sensitivity and resolution, MSI has been instrumental in profiling element-specific signatures in a range of materials and has propelled forward studies in fields such as medicine, biology, and material science.^4−7^

Three-dimensional (3D) MSI advances beyond 2D MSI by providing the ability to profile the depth of biological samples, thereby enabling the mapping of biomolecules in three dimensions within tissues and organs. It is extremely challenging for mass spectral imaging to map molecular composition in 3D. MS imaging nowadays is performed almost exclusively in 2D mode or by reconstruction of 3D objects by software-stacking of many 2D results.^8^ For the realization of 3D software reconstructed MSI, tissues or organs are first sectioned in series. These sections are subsequently imaged in two dimensions and reconstructed into three-dimensional images. Measurement techniques used for the above-mentioned analyses are usually desorption electrospray ionization (DESI),^9^ nanospray desorption electrospray ionization (Nano-DESI),^10^ laser ablation electrospray ionization (LAESI),^11^ and matrix-assisted laser desorption ionization (MALDI).^12^

Most frequently, 3D-reconstructed MSI studies are performed with the use of matrix-assisted laser desorption/ionization mass spectrometry (MALDI MS).^13^ MALDI MS has some limitations, such as a crowded chemical background in the low-mass region if low resolving power instrumentation is used^14^ or not optimal ionization of neutral organic compounds.^8^ Additionally, commercial instruments suitable for MALDI MSI do not have the possibility of sample freezing.

There are also reports of 3D-reconstructed MSI results made with the use of DESI.^9^ One of the greatest advantages of sample preparation prior to analysis. Until recently, a major limitation of DESI was its low resolution, typically ranging from tens to hundreds of micrometers.^15^ However, the introduction of nano-DESI allowed for achieving resolution up to 6 μm.^16^ Controlling the distance between the nano-DESI probe and the sample surface is critical to achieving high resolution but is also technically challenging to maintain, especially for thinner tissue sections.^17^ Despite significant improvements, the ionization efficiency in nano-DESI may still be insufficient for some types of analysis, particularly when analyzing complex biological matrices. A common issue with nano-DESI is the stability of solvent flow, which is dependent on the inlet vacuum of the mass spectrometer. A major limitation of DESI is the small depth of penetration of liquid into the sample, which is typically limited to a few micrometers. For example, DESI imaging did not cause physical damage to the underlying cells on the algal surfaces.^18^ Although the sample removal rate of DESI is similar to that of secondary ion mass spectrometry (SIMS)^19^ analytes are removed selectively, which does not allow depth profiling for three-dimensional imaging - a capability that can be achieved with SIMS^20^ and LAESI.^21^

Methods reported in the literature as capable of direct, nonsoftware reconstructed 3D MS imaging include SIMS,^22^ which uses energetic ion bombardment to erode the surface of a sample.^23^ SIMS-based time-of-flight (ToF) MSI is capable of depth profiling molecular content with 10 nm depth resolution. There are also reports of 60 nm depth profiling, but heavy molecular fragmentation is observed.^24,25^ In 2020, Zhang et al.^23^ presented Cryo-OrbiSIMS for 3D molecular imaging of a frozen bacterial biofilm. It was possible to perform depth profiling by removing up to 30–50 μm of the object. With a shallow ablation depth, it is impossible to obtain a deep profile of macroscopic objects. The necessity of a high vacuum inside the system prevents the analysis of biological objects without risking deformation. Another approach was published with the use of an extreme ultraviolet (EUV) laser.^26^ The authors achieved submicrometer resolutions, achieving a lateral resolution of 75 nm and a depth resolution of 20 nm.

The laser ablation remote atmospheric pressure photoionization/chemical ionization (LARAPPI/CI) 2D and 3D MSI platform presented in this work provides a solution to various problems associated with 3D MSI, such as reconstruction of 2D to 3D models, migration of metabolites, sample dehydration, evaporation of metabolites, a large volume of ablated material, and occurrence of artifacts from mechanical section preparations. Also, this allows for control shape and depth of the ablated surface, providing 3D results. The proposed solution should also be compatible with mass spectrometers that use common electrospray ionization (ESI) ion sources; it is not compatible with instruments with vacuum ion sources. The integrated 3D distance sensor acts as a surface profilometer, allowing for ablation control during the experiments.

## Results and Discussion

Direct three-dimensional MSI is considered one of the most useful analytical methods today. The possibility of detection of hundreds of compounds within a single microscopic space (voxel) on the surface (2D MSI) or inside (3D MSI) of the studied sample, and then their localization in the object, gives unlimited possibilities to biologists, biochemists, and material chemists. To perform such analysis, the method is capable of removing relatively large volumes of material in a strictly controlled manner, and also on the microscale, and quickly transferring this material to the mass spectrometer. Precise removal of entire layers of material is needed to access the lower parts or layers of the analyzed samples.

### Description of the Experimental Setup

**Figure 1:**
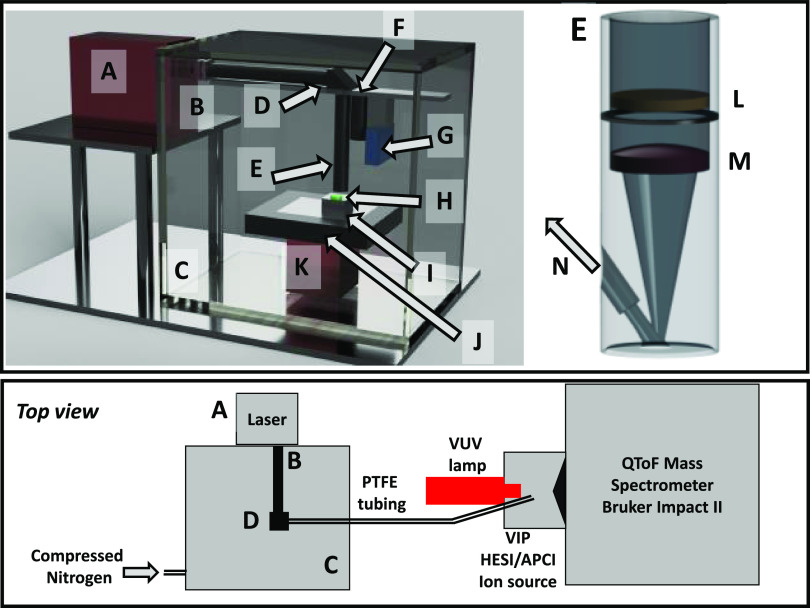
Schematic of the LARAPPI/CI MSI system. The upper panel presents a perspective view of the system without a mass spectrometer; the lower panel contains a simplified top view of the whole system. (A) OPO laser, (B) sapphire window, (C) pressure chamber, (D) gold mirror assembly, (E) laser focus assembly, (F) camera, (G) distance sensor, (H) sample, (I) sample stage with Peltier module and water block, (J) *XY* high-speed stage, (K) *Z* vertical stage, (L) diffractive optical element, (M) aspherical ZnSe lens, and (N) ablated material port connected via PTFE tubing to the ion source.

In the LARAPPI/CI MSI system, computer-controlled ablation takes place in a pressure chamber working at atmospheric pressure. The sample is placed on a sample stage (Figure 1I) with a built-in Peltier cooling plate that allows for freezing of the sample for the experiment. The temperature-controlled sample stage is mounted on a motorized high-speed *XY* stage. The pulsed beam from the OPO laser (Figure 1A) of 2930 nm wavelength expanded 3.75 times is redirected toward the sample stage by a gold-plated mirror (Figure 1D), goes through the diffractive optical element, and is focused onto the sample surface by a 50 mm focal length aspherical lens.

The system also contains a camera and a high-precision distance sensor acting as a profilometer. During imaging, the laser focal point remains fixed in space, while the sample (Figure 1H) is moved by the computer-controlled *XY*- and *Z*-stages. A specially designed gas funnel (Figure 1E), also a focusing assembly, is placed on the ablation site. The overpressure in the chamber drives dry nitrogen gas with ablated material to the ion source. The samples are kept at subzero temperatures during analysis to keep their shape unchanged and prevent metabolites from migration.

LARAPPI/CI uses diffractive optical elements produced by HOLO/OR for the generation of a square-focused beam with a flat top profile.^27^ The difference in ablation crater shape for the modified beam can be seen in the laser printer paper test results shown in Supporting Information S1.

**Figure 2:**
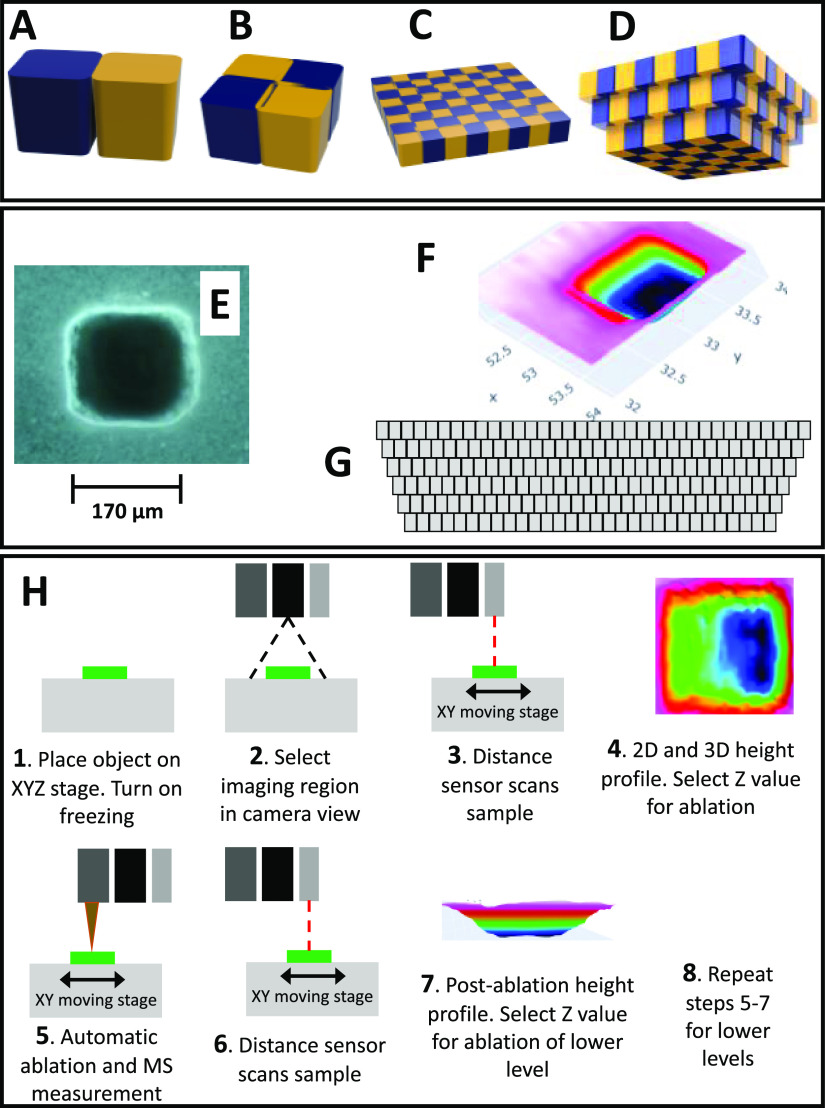
Renderings of 3D models of ablated voxel spaces: (A) not optimal arrangement of two voxels (notice that the lower part of space between voxels is not matching); (B) oversampling of four voxels allows for complete ablation of space; (C) example of oversampling arrangement for one layer of 8 × 8 voxels array; (D) inverted pyramid arrangement for oversampled voxels for three-dimensional ablation of material. The lower panel presents: (E) optical microscope image of a single voxel ablated in an agar gel with 20 laser pulses; (F) profilometer scan result of the ablated space of agar gel for a 7 × 7 × 1 voxel array with 140 × 140 μm resolution (20 laser pulses per voxel). (G) simplified inverted pyramid arrangement of voxels in kiwi and red radish 3D MSI experiments as seen from the side of the object; (H) 2D (steps 1–5) and 3D (steps 1–8) MSI workflow.

A graphical representation of the volume removed by this setup is presented in Figure 2A (blue or yellow). As can be seen, a single-voxel shape is part of a pentahedron with rounded side edges. As can be seen in Figure 2A, using laser ablation to remove these shapes leaves some material between them. Oversampling with stacked voxels as presented in Figure 2B improves material removal during multivoxel ablations. To perform ablation without the removal of upper layers due to the fixed shape of the focused laser beam, we introduced an inverted pyramid ablation scheme (Figure 2D).

Single point ablation with 20 laser pulses of agar gel enriched for opacity in titanium dioxide nanopowder produced the desired square shape with rounded edges (170 × 170 μm size) as shown in the optical photograph in Figure 2E. Optimization of oversampling (shown in Supporting Information S7) suggested that 140 μm voxel-center-to-voxel-center (please see Figure 2B–D) produced optimal results with a relatively flat bottom of 7 × 7 × 1 voxel ablation area (depth 330 μm) as judged by optical microscope observation and also profiling with a distance sensor (Figure 1F). Additional ablation results of agar gel of 11 × 7 (*X* × *Y*, top-level resolution) space are shown in Supporting Information S2. Figure 2G presents a side view of the inverted pyramid ablation scheme. As can be seen, the layers are ablated with the number of lines in the *X* and *Y* axes reduced by 1 for every ablation layer below the first one (example: first-layer resolution, 40 × 40, second 39 × 39, etc.). Additionally, the voxel pattern of each lower layer is shifted to the center of the ablated region. This ablation scheme allows avoidance of ablation of walls of upper layers, which is one of the biggest problems in 3D MSI.^11^ The undesirable ablation of upper layers in SIMS was partially solved by the utilization of two ion beams, the first beam ejects atoms, molecules, and secondary ions from the surface, while the second beam sputters the already analyzed surface to create a new plane for imaging the next layer.^28^ Theoretically, a similar solution could be possible for laser systems; however, for complete removal of material, it would still require advanced beam shaping and beam direction control, making it much more complicated and expensive than the solution presented in this work. Each 3D MSI experiment presented below was performed in an inverted pyramid mode (Figure 2D,G). To present the procedure for 2D and 3D imaging in an easily understandable form, a workflow is presented in Figure 2I.

### Optimization of LARAPPI/CI Working Conditions

To provide the highest sensitivity for the detection of biological compounds in microscopic-sized ablation voxels, we have modified the Bruker VIP HESI ion source in an APCI configuration to introduce a dopant-assisted APPI ionization by using a vacuum ultraviolet (VUV) lamp producing light of a wavelength in the 110–160 nm range. This ion source irradiates a pulsed stream of ablated material from the chamber that generates low-temperature plasma in the gas phase through direct interaction with biological compounds and, most likely, with toluene vapor from the toluene-methanol mixture pumped to the APCI needle by the HPLC pump (Figure 1 bottom panel). Tests of this ion source showed that ions forming from ablated material are the deprotonated ones of the [M-H]^−^ formula, which is in accordance with results from both APPI and APCI ion sources.^29−31^ During preliminary experiments, we tested electrospray ionization (ESI), atmospheric pressure chemical ionization (APCI), and atmospheric pressure photoionization (APPI) ionization in both positive and negative ion detection modes. After the optimal ionization type, APCI/APPI, the liquid composition, and the flow rate were also optimized. All optimizations were carried out under conditions similar to the operating mode of the MSI system; therefore, all tests were based on laser ablation of frozen agar gel containing test compounds, each compound at a concentration of 100 μg/mL. The ablated volumes calculated for each voxel were approximately 6.6 × 10^–6^ mL, which equates to 660 pg of each test compound. The test compounds were polar to medium polar biological compounds: ribose, histidine, thymidine, and uracil. The results of the tests mentioned are shown in Supporting Information S4 and S5 and Table S1. As can be concluded from the data in Table S1 and Supporting Information S5, the highest S/N ratios were obtained in the APCI/APPI negative ion mode. All compounds were detected in the APCI, APCI/APPI positive mode, and ESI/APPI negative modes. ESI/APPI results were of lower S/N compared to APCI/APPI in both positive and negative modes, which suggests that the intensity of interactions of charged droplets emitted by the ESI needle with ablated plume material is much lower compared to interactions of plume with more abundant charged gaseous species emitted in the APCI modes.

As the results shown above suggest, the best results were obtained for APCI/APPI. Optimization of the solvent mixture pumped into our modified APCI/APPI ion source was performed in a manner similar to that in the above-described experiments using agar gel laser ablation. In total, eight combinations of solvents (acetonitrile, methanol, acetone), with various dopants (toluene, formic acid) based on the literature were tested (Supporting Information S3) at different flow speeds (0.01 to 0.3 mL/min).^32−38^ The highest average S/N value for all four compounds was for 1% toluene in methanol at a flow rate of 200 μL/min flow rate. In conclusion, the optimal ionization conditions in our MSI setup are the combination of APCI with DA-APPI.

### Examples of Three-Dimensional Results from the LARAPPI/CI MSI System

**Figure 3:**
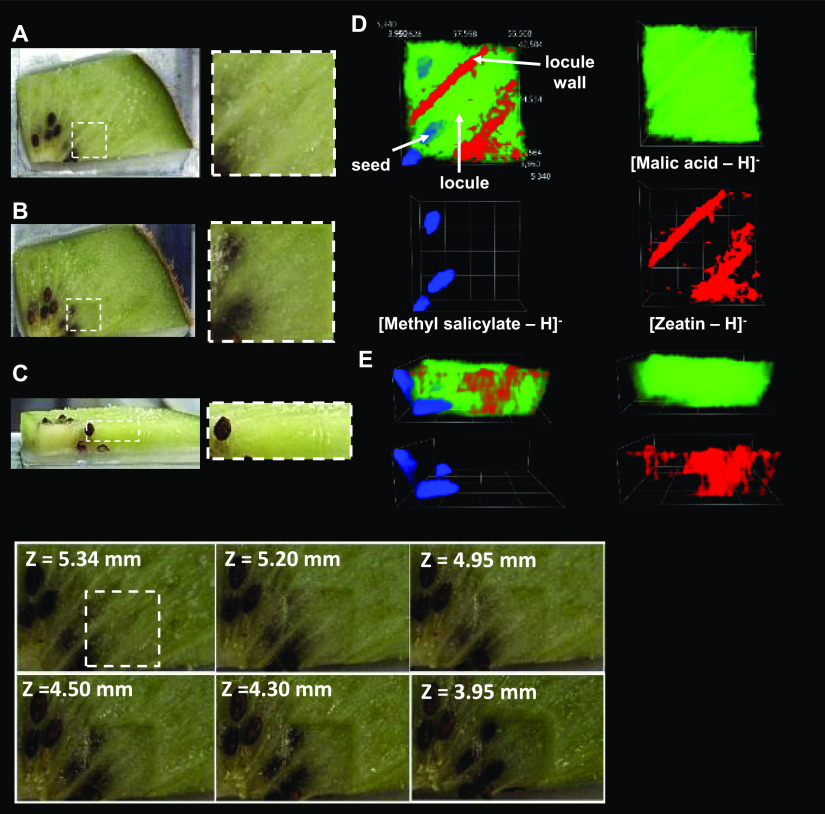
Photographs of the object studied (kiwi fruit cross section) and selected 3D MSI results. (A) Optical photographs of the starting object and analysis region marked with a white dashed line; (B) optical photographs of the postanalysis object; (C) side view of the object; (D) 3D MSI ion images for three ions represented by different colors–top views; (E) side views of 3D ion images of ions of different colors. The intensity of an ion signal is represented by the opacity of the 3D cloud within a given color. The bottom panel contains optical photographs of the preablation region and after the ablation steps. The *Z*-value was measured with the precision distance sensor.

The tissue of the kiwi fruit was selected as a real-life biological test for the 3D possibilities of the LARAPPI/CI MSI system. The experiment region was selected as shown in Figure 3A,B due to the placement of the seeds under a thick layer of parenchyma. Published MSI studies of radish taproot, kiwi fruit, and human kidney tissue were carried out only in 2D imaging mode.^39−42^ In the case of kiwi fruit, direct 3D MSI was performed in 6 steps or layers. The highest level of ablation was ablated at a resolution of 140 × 140 μm resolution in a 35 × 35 (*X* × *Y*) voxel arrangement with a total depth after the six ablation steps of 1.43 mm. The total number of voxels ablated in the experiment was 6355, while the ablation time took only 3 h and 20 min plus 3 min after each level for the ablation region profiling. The entire experiment removed approximately 25–30 mm^3^, volume of tissue material, which is a unique characteristic of this system.

**Figure 4:**
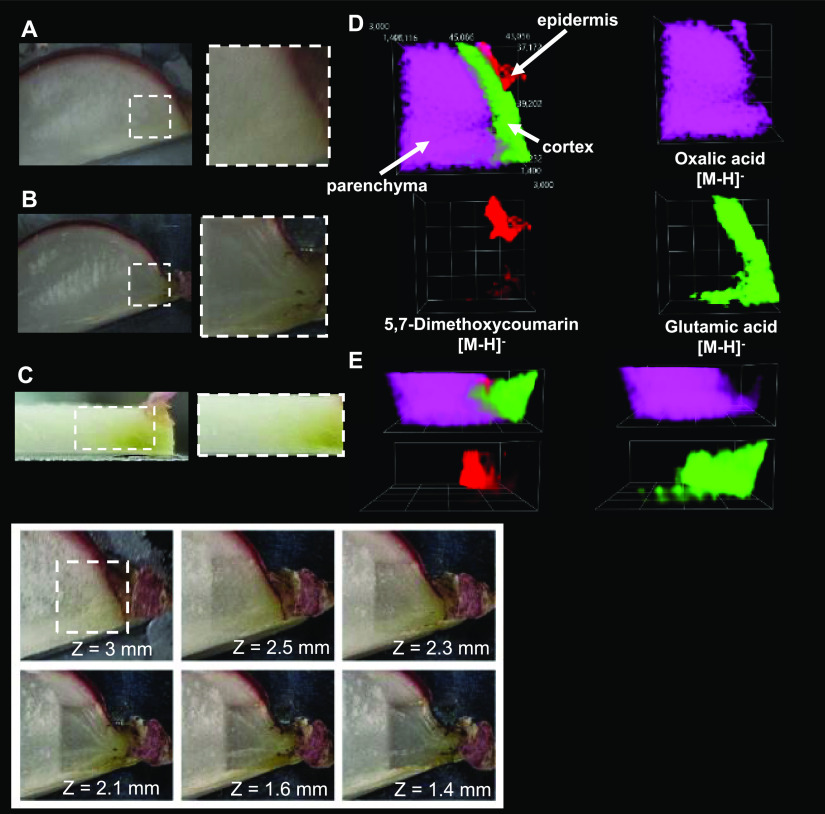
Photographs of the object studied (red radish cross section) and selected 3D MSI results. (A) Optical photographs of the starting object and analysis region marked with a white dashed line; (B) optical photographs of the postanalysis object; (C) side view of the object; (D) 3D MSI ion images for three ions represented by different colors—top views; (E) side views of 3D ion images of ions of different colors. The intensity of an ion signal is represented by the opacity of the 3D cloud within a given color. The bottom panel contains optical photographs of the preablation region and after the ablation steps. The *Z*-value was measured with the precision distance sensor.

**Figure 5:**
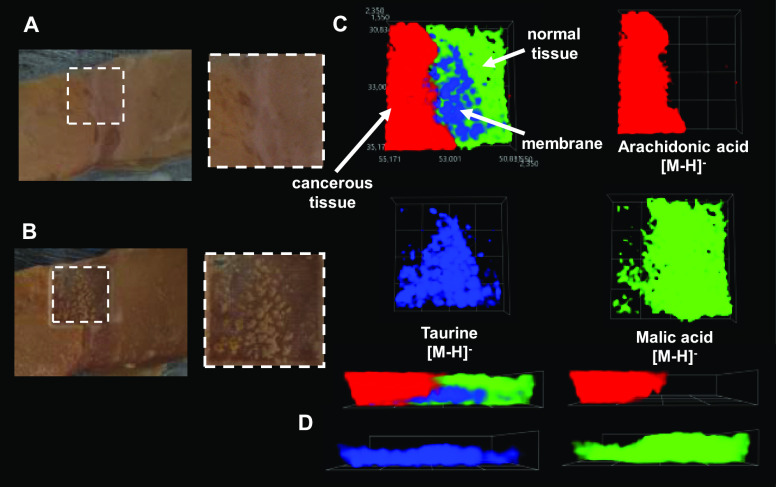
Photographs of the object studied (human kidney tissue) and selected 3D MSI results. (A) Optical photographs of the starting object and analysis region marked with a white dashed line; (B) optical photographs of the postanalysis object; (C) side view of the object; (D) 3D MSI ion images for three ions represented by different colors–top views; (C, D) side views of 3D ion images of ions of different colors. The intensity of an ion signal is represented by the opacity of the 3D cloud within a given color.

It should be noted that the experiment examples shown in each figure with MSI results (Figures 3 to 5) contain ion distributions of just three ions out of hundreds for the sake of clarity. The search provides information that kiwi fruit tissue was studied by MALDI MSI that provided information on differences in compound distribution in pulp, skin, and seeds.^43^ In the case of our experiment, one of the 3D-imaged compounds in this fruit was malic acid (Figure 3D,E, green color), widely present as a side product of carbohydrate metabolism in various tissues.^44^ The methyl salicylate found in the seeds of kiwi fruit (Figure 3D,E, blue color) was previously found in blended kiwi pulp.^45^ It is said to play a role in signaling, and when demethylated to salicylic acid, exhibits anti-inflammatory properties.^46,47^ Zeatin, found in the locule walls of the kiwi fruit (Figure 3D,E, red color), is a cytokinin responsible for the regulation of plant growth.^48^

3D MSI of a cross section of red radish is the next example of possibilities of the LARAPPI/CI MSI platform. The compounds identified in the root of the radish stem originate from LC-MS and MS/MS analyses conducted by our team (Supporting Information S6). Oxalic acid (4D,E, purple) is a compound commonly found in almost all plants. The most prominent function suggested for oxalic acid in plants is the retention of ions, specifically calcium, that form calcium oxalate crystals.^49^ It has also been associated with plant growth, development regulation, and stress responses.^50^ 5,7-Dimethoxycoumarin (Figure 4D,E, red color) has been previously identified in the epidermis of various plants, such as *Citrus aurantifolia* peel, and has been reported to possess antioxidant properties.^51^ Glutamic acid (Figure 4D,E, green color) was found in plants to promote callus formation,^52^ regulate nitrogen metabolism, serve as a precursor to other amino acids, and is also a building block for proteins.^53^

2D MSI of a red radish root (*Raphanus sativus*) was conducted in 2014 by Seaman et al. by MALDI-MS imaging. The research was focused on the metabolism of nitrogen into amino acids.^54^ A different work from 2015 utilized laser ablation and solvent capture by aspiration (LASCA) with an electrospray ion source. The ablation process with an ablation depth of 10–25 μm and volume of approximately 1 nl per pixel allowed differentiation of the spatial distributions in varying structures of the root proved possible, however, the images were still two-dimensional, as stated previously.^55^

Human kidney tissue with visible cancer and normal regions was studied with a 3D MSI system. The identification of compounds found in kidney tissue was performed using previous research. Arachidonic acid (Figure 5D,E, red color) is linked to pro-inflammatory activity in kidney tissue, being released as a result of cell stress. Arachidonic acid then acts as a precursor to bioactive mediators that can lead to renal dysfunction.^56,57^ The ways taurine levels (Figure 5D,E, blue color) influence renal processes are many, such as ion reabsorption, antioxidant properties, impact of blood flow, cell apoptosis, and more.^58,59^ The taurine transporter gene is down-regulated by the chemotherapeutic agent cisplatin.^60^ Malic acid (Figure 5D,E, green color), widely present as a side product of carbohydrate metabolism in various tissues, has also been previously identified in kidney tissue.^61,62^

2D mass spectrometry imaging of both normal tissue and cancerous tissue of the human kidney has previously been performed for lipidomic, drug, and drug metabolite spatial distribution or proteomic analyses. Zhang et al. have used desorption electrospray ionization mass spectrometry imaging (DESI-MSI) to study metabolic profiles of renal oncocytoma, renal cell carcinoma (RCC), and healthy tissue to utilize the data obtained data for building prediction models.^63^ DESI-MSI has also been used as a potential prognostic tool for RCC by Vijayalakshmi et al.^64^ Other methods, such as MALDI-MSI for protein and lipid profiling^65^ and SALDI-MSI for cancer biomarker identification,^66^ have also been performed. Even despite successful results of 2D MSI, two-dimensional methods of profiling can prove unreliable in a tissue of highly varied structure and, therefore, chemical composition, which can only become known by three-dimensional analysis.

## Materials and Methods

All chemicals were analytical reagent grade. All solvents were of LC-MS purity, except water (18 MΩcm water produced locally). Steel plates of 4.5 cm × 3.5 cm size used as sample plates were machined from H17 stainless steel of 0.8 mm thickness. Optical photographs and size/depth measurement results of ablation shapes were obtained with a motorized microscope built locally with three motorized stages in *XYZ* configuration (ThorLabs MTS50), DeltaOptical USB 3.0 camera (DLT Cam Pro 14 MPix) with a 2.5x InfiniFlex HD Compact Lens, and microscope light ring. Syringe filters (PTFA membrane, 0.2 μm-pore) were purchased from Merck Poland.

### LARAPPI/CI System Setup

A Nd/YAG-pumped, OPO laser (Opolette HE 2940 model, factory tuned to 2930 ± 1 nm); Opotek, Carlsbad, CA, USA) with a pulse length shorter than 7 ns generated mid-IR laser pulses with a maximum repetition frequency of 20 Hz. The pulse energy measured before the diffractive element was 3.5 mJ (measured using a pyroelectric energy meter, PE25-SH-V2; Ophir, Logan, UT, USA).

The LARAPPI/CI system is based on an airtight chamber, as shown in Figure 1. The chamber (C) is pressurized with nitrogen gas to produce a nitrogen stream of 10 L/min. The sample is placed on a 50 × 50 mm sample stage (I) made of aircraft-grade aluminum alloy; under it, there is a Peltier cooling plate (TE-127–1.4–1.5; TE Technology, Traverse City, MI, USA) that maintains the sample at temperatures as low as −18 °C. The heat generated from the Peltier element is removed using circulating water and an external radiator (not shown in Figure 1).

The temperature-controlled sample stage is mounted on a motorized high-speed *XY*-stage (Figure 1J; MLS203, Thorlabs, Sweden). The pulsed beam from the OPO laser (Figure 1A) enters the sample chamber (Figure 1C) through a 1″ sapphire window (Figure 1B) with both sides with AR-coatings (Thorlabs, Sweden), is then expanded with CaF_2_ plano-concave lens of f = −40.0 mm, and collimated with CaF_2_ plano-convex lens of *f* = 150 mm, both are AR-coated for 2–5 μm (not shown in Figure 1). The expanded laser beam is redirected toward the sample stage by a 1 in. gold-plated mirror (Figure 1D; Thorlabs, Sweden) and goes through a diffractive optical element (HM-396, Holo-OR Ltd. Israel), and is focused onto the sample surface by a 50 mm focal length aspherical ZnSe lens with AR coatings (both in Figure 1E, Thorlabs, Sweden).

The optical assembly and also the camera (Figure 1F; FLIR Blackfly S, Color Camera, 6 MPix, Sony IMX178 sensor) with a lens (12 mm C Series Fixed Focal Length Lens, Edmund Optics, UK) and distance sensor (Baumer OM70; Figure 1G) are mounted on locally machined precision aluminum rails and are in a fixed configuration; the only moving parts are *XY* (Figure 1J) and *Z* (Figure 1K) stages that are mounted on a precision aluminum 15 mm plate mounted at the bottom of the pressure chamber. During imaging, the laser focal point remains fixed in space, whereas the sample (Figure 1H) is moved by the computer-controlled *XY*- and *Z*-stages. A specially designed gas funnel (Figure 1E) is also a focusing assembly and is connected to a 6/4 mm (O.D/I.D.) PTFE tube. The gas funnel bottom surface is placed over (∼4 mm) the laser ablation site. The overpressure in the chamber drives a 10 L/min nitrogen gas flow through the tube. The nitrogen, chamber lighting, and cooling systems are connected through relays and controlled by a control program.

The laser ablation plumes are entrained into the gas and transported to the modified ion source (Bruker VIP HESI in the APCI configuration) of the Bruker Impact II mass spectrometer. The outlet end of the PTFE tube is mounted at an angle of 30° to the axis of the spectrometer inlet. The ion source also has a VUV source (Hamamatsu L12542) mounted axially to the MS sampling cone inside the ion source. A binary HPLC pump (Agilent G1312A) provided a steady flow of a solvent mixture (1% toluene in methanol; 200 μL/min) to the APCI needle.

Samples are kept at −18 °C during analysis by the Peltier module. The spatial resolution is typically 140 μm with applied oversampling (Figure 2). Each pixel/voxel in 3D MSI experiments was exposed to the laser for 500 ms, at a laser pulse repetition rate of 20 Hz. The delays between pixels were 1000 ms. Between pixels, the sample stage moved at a speed of 50 mm/s. The time delay between lines was 3 s. Time-synchronization of laser pulses with signals recorded by the mass spectrometer is aided by an ethernet-based time server.

Each 3D experiment was carried out in an inverted pyramid scheme (Figure 2D) with the following procedure (Figure 2H):

1. Calibration of the MS instrument.
2. Sample scanning with a distance sensor, generation, and user-analysis of the 2D and 3D profile of the object’s top side shape.
3. Setting the ablation region with the camera image.
4. Setting the *Z* level for the first ablation level.
5. Ablation of the layer with the recording of MS or MS + bbCID data.
6. Profiling of ablated object with a distance sensor; visual inspection with the camera image.
7. Setting the *Z* level for the next ablation, etc. It must be noted that the *XY* ablation area of the lower layers is set automatically by software with the same resolution as the first layer but with an ablation area smaller by one *X* and one *Y* row and also centered.

## Conclusions

We created a laser ablation remote atmospheric pressure photoionization/chemical ionization (LARAPPI/CI) platform coupled to an ultrahigh resolution quadrupole-time-of-flight (QToF) mass spectrometer. The optics of this system is based on a mid-IR laser and diffractive optical element. We have shown a novel approach, the inverted pyramid ablation scheme that is suitable for the removal of the layers required for three-dimensional MSI. Various optimizations of the MSI system are shown, and the most important one is the modified APCI/dopant-assisted-APPI ion source. The MSI solution was used for the direct three-dimensional (3D) mass spectrometry imaging (MSI) of metabolites in human and plant tissues.

## Data Availability

The data sets generated during and/or analyzed during the current study are available from the corresponding author upon request and in the RepOD open data repository (doi: 10.18150/WWFOHP and 10.18150/UOR5P1).

## Supporting Information Available

(Figures S1–S10) Additional details on the optimization of the MSI setup, parameters for 3D MSI experiments, LC-MS experimental details, and results (PDF)

## References

[ref1] Norris J. L.; Caprioli R. M. Analysis of Tissue Specimens by Matrix-Assisted Laser Desorption/Ionization Imaging Mass Spectrometry in Biological and Clinical Research. Chem. Rev. 2013, 113 (4), 2309–2342. 10.1021/cr3004295. 23394164 PMC3624074

[ref2] Bednařík A.; Prysiazhnyi V.; Bezdeková D.; Soltwisch J.; Dreisewerd K.; Preisler J. Mass Spectrometry Imaging Techniques Enabling Visualization of Lipid Isomers in Biological Tissues. Anal. Chem. 2022, 94 (12), 4889–4900. 10.1021/acs.analchem.1c05108. 35303408

[ref3] Buchberger A. R.; DeLaney K.; Johnson J.; Li L. Mass Spectrometry Imaging: A Review of Emerging Advancements and Future Insights. Anal. Chem. 2018, 90 (1), 240–265. 10.1021/acs.analchem.7b04733. 29155564 PMC5959842

[ref4] Buchberger A. R.; DeLaney K.; Johnson J.; Li L. Mass Spectrometry Imaging: A Review of Emerging Advancements and Future Insights. Anal. Chem. 2018, 90 (1), 240 10.1021/acs.analchem.7b04733. 29155564 PMC5959842

[ref5] Wang T.; Cheng X.; Xu H.; Meng Y.; Yin Z.; Li X.; Hang W. Perspective on Advances in Laser-Based High-Resolution Mass Spectrometry Imaging. Anal. Chem. 2020, 92 (1), 543–553. 10.1021/acs.analchem.9b04067. 31755699

[ref6] Dunham S. J. B.; Ellis J. F.; Li B.; Sweedler J. V. Mass Spectrometry Imaging of Complex Microbial Communities. Acc. Chem. Res. 2017, 50 (1), 96–104. 10.1021/acs.accounts.6b00503. 28001363 PMC5244435

[ref7] Neumann E. K.; Comi T. J.; Spegazzini N.; Mitchell J. W.; Rubakhin S. S.; Gillette M. U.; Bhargava R.; Sweedler J. V. Multimodal Chemical Analysis of the Brain by High Mass Resolution Mass Spectrometry and Infrared Spectroscopic Imaging. Anal. Chem. 2018, 90 (19), 11572–11580. 10.1021/acs.analchem.8b02913. 30188687 PMC6168410

[ref8] Li D.; Qian Y.; Yao H.; Yu W.; Ma X. DeepS: Accelerating 3D Mass Spectrometry Imaging via a Deep Neural Network. Anal. Chem. 2023, 95 (29), 10879–10886. 10.1021/acs.analchem.2c05785. 37427961

[ref9] Eberlin L. S.; Ifa D. R.; Wu C.; Cooks R. G. Three-Dimensional Vizualization of Mouse Brain by Lipid Analysis Using Ambient Ionization Mass Spectrometry. Angew. Chem. 2010, 122 (5), 885–888. 10.1002/ange.200906283. PMC2958060 20041465

[ref10] Lanekoff I.; Burnum-Johnson K.; Thomas M.; Cha J.; Dey S. K.; Yang P.; Prieto Conaway M. C.; Laskin J. Three-Dimensional Imaging of Lipids and Metabolites in Tissues by Nanospray Desorption Electrospray Ionization Mass Spectrometry. Anal Bioanal Chem. 2015, 407 (8), 2063–2071. 10.1007/s00216-014-8174-0. 25395201 PMC5044879

[ref11] Nemes P.; Barton A. A.; Vertes A. Three-Dimensional Imaging of Metabolites in Tissues under Ambient Conditions by Laser Ablation Electrospray Ionization Mass Spectrometry. Anal. Chem. 2009, 81 (16), 6668–6675. 10.1021/ac900745e. 19572562

[ref12] Andersson M.; Groseclose M. R.; Deutch A. Y.; Caprioli R. M. Imaging Mass Spectrometry of Proteins and Peptides: 3D Volume Reconstruction. Nat. Methods 2008, 5 (1), 101–108. 10.1038/nmeth1145. 18165806

[ref13] Paine M. R. L.; Liu J.; Huang D.; Ellis S. R.; Trede D.; Kobarg J. H.; Heeren R. M. A.; Fernández F. M.; MacDonald T. J. Three-Dimensional Mass Spectrometry Imaging Identifies Lipid Markers of Medulloblastoma Metastasis. Sci. Rep. 2019, 9 (1), 2205 10.1038/s41598-018-38257-0. 30778099 PMC6379434

[ref14] Nizioł J.; Ossoliński K.; Ossoliński T.; Ossolińska A.; Bonifay V.; Sekuła J.; Dobrowolski Z.; Sunner J.; Beech I.; Ruman T. Surface-Transfer Mass Spectrometry Imaging of Renal Tissue on Gold Nanoparticle Enhanced Target. Anal. Chem. 2016, 88 (14), 7365–7371. 10.1021/acs.analchem.6b01859. 27329270

[ref15] Eberlin L. S.; Ifa D. R.; Wu C.; Cooks R. G. Three-Dimensional Vizualization of Mouse Brain by Lipid Analysis Using Ambient Ionization Mass Spectrometry. Angew. Chem., Int. Ed. 2010, 49 (5), 873–876. 10.1002/anie.200906283. PMC2958060 20041465

[ref16] Unsihuay D.; Hu H.; Qiu J.; Latorre-Palomino A.; Yang M.; Yue F.; Yin R.; Kuang S.; Laskin J. Multimodal High-Resolution Nano-DESI MSI and Immunofluorescence Imaging Reveal Molecular Signatures of Skeletal Muscle Fiber Types. Chem. Sci. 2023, 14 (15), 4070–4082. 10.1039/D2SC06020E. 37063787 PMC10094364

[ref17] Jiang L. X.; Hilger R. T.; Laskin J. Hardware and Software Solutions for Implementing Nanospray Desorption Electrospray Ionization (Nano-DESI) Sources on Commercial Mass Spectrometers. J. Mass Spectrom 2024, 59 (7), e5065 10.1002/jms.5065. 38866597 PMC11330693

[ref18] Lane A. L.; Nyadong L.; Galhena A. S.; Shearer T. L.; Stout E. P.; Parry R. M.; Kwasnik M.; Wang M. D.; Hay M. E.; Fernandez F. M.; Kubanek J. Desorption Electrospray Ionization Mass Spectrometry Reveals Surface-Mediated Antifungal Chemical Defense of a Tropical Seaweed. Proc. Natl. Acad. Sci. U. S. A. 2009, 106 (18), 7314–7319. 10.1073/pnas.0812020106. 19366672 PMC2678663

[ref19] Salter T. L.; Green F. M.; Gilmore I. S.; Seah M. P.; Stokes P. A Comparison of SIMS and DESI and Their Complementarities. Surf. Interface Anal. 2011, 43 (1–2), 294–297. 10.1002/sia.3412.

[ref20] Wucher A.; Cheng J.; Winograd N. Protocols for Three-Dimensional Molecular Imaging Using Mass Spectrometry. Anal. Chem. 2007, 79 (15), 5529–5539. 10.1021/ac070692a. 17583913

[ref21] Nemes P.; Barton A. A.; Vertes A. Three-Dimensional Imaging of Metabolites in Tissues under Ambient Conditions by Laser Ablation Electrospray Ionization Mass Spectrometry. Anal. Chem. 2009, 81 (16), 6668–6675. 10.1021/ac900745e. 19572562

[ref22] Seeley E. H.; Caprioli R. M. 3D Imaging by Mass Spectrometry: A New Frontier. Anal. Chem. 2012, 84 (5), 2105–2110. 10.1021/ac2032707. 22276611 PMC3296907

[ref23] Zhang J.; Brown J.; Scurr D. J.; Bullen A.; Maclellan-Gibson K.; Williams P.; Alexander M. R.; Hardie K. R.; Gilmore I. S.; Rakowska P. D. Cryo-OrbiSIMS for 3D Molecular Imaging of a Bacterial Biofilm in Its Native State. Anal. Chem. 2020, 92 (13), 9008–9015. 10.1021/acs.analchem.0c01125. 32460495

[ref24] Gunnarsson A.; Kollmer F.; Sohn S.; Höök F.; Sjövall P. Spatial-Resolution Limits in Mass Spectrometry Imaging of Supported Lipid Bilayers and Individual Lipid Vesicles. Anal. Chem. 2010, 82 (6), 2426–2433. 10.1021/ac902744u. 20163177

[ref25] Lechene C.; Hillion F.; McMahon G.; Benson D.; Kleinfeld A. M.; Kampf J. P.; Distel D.; Luyten Y.; Bonventre J.; Hentschel D.; Park K.; Ito S.; Schwartz M.; Benichou G.; Slodzian G. High-Resolution Quantitative Imaging of Mammalian and Bacterial Cells Using Stable Isotope Mass Spectrometry. J. Biol. 2006, 5 (6), 20–30. 10.1186/jbiol42. 17010211 PMC1781526

[ref26] Kuznetsov I.; Filevich J.; Dong F.; Woolston M.; Chao W.; Anderson E. H.; Bernstein E. R.; Crick D. C.; Rocca J. J.; Menoni C. S. Three-Dimensional Nanoscale Molecular Imaging by Extreme Ultraviolet Laser Ablation Mass Spectrometry. Nat. Commun. 2015, 6 (1), 6944 10.1038/ncomms7944. 25903827 PMC4423227

[ref27] Bai H.; Manni J. G.; Muddiman D. C. Transforming a Mid-Infrared Laser Profile from Gaussian to a Top-Hat with a Diffractive Optical Element for Mass Spectrometry Imaging. J. Am. Soc. Mass Spectrom. 2023, 34 (1), 10–16. 10.1021/jasms.2c00203. 36542595 PMC9975536

[ref28] Passarelli M. K.; Pirkl A.; Moellers R.; Grinfeld D.; Kollmer F.; Havelund R.; Newman C. F.; Marshall P. S.; Arlinghaus H.; Alexander M. R.; West A.; Horning S.; Niehuis E.; Makarov A.; Dollery C. T.; Gilmore I. S. The 3D OrbiSIMS-Label-Free Metabolic Imaging with Subcellular Lateral Resolution and High Mass-Resolving Power. Nat. Methods 2017, 14 (12), 1175–1183. 10.1038/nmeth.4504. 29131162

[ref29] Dousty F.; Sc M. The Use of Dopants in Atmospheric Pressure Ionization Sources of Mass Spectrometers; University of British Columbia 2015.

[ref30] Tubaro M.; Marotta E.; Seraglia R.; Traldi P. Atmospheric Pressure Photoionization Mechanisms. 2. The Case of Benzene and Toluene. Rapid Commun. Mass Spectrom. 2003, 17 (21), 2423–2429. 10.1002/rcm.1208. 14587089

[ref31] McEwen C. N.; Larsen B. S. Ionization Mechanisms Related to Negative Ion APPI, APCI, and DART. J. Am. Soc. Mass Spectrom. 2009, 20 (8), 1518–1521. 10.1016/j.jasms.2009.04.010.

[ref32] Rhourri-Frih B.; Chaimbault P.; Claude B.; Lamy C.; André P.; Lafosse M. Analysis of Pentacyclic Triterpenes by LC–MS. A Comparative Study between APCI and APPI. Journal of Mass Spectrometry 2009, 44 (1), 71–80. 10.1002/jms.1472. 18946879

[ref33] Himmelsbach M.; Buchberger W.; Reingruber E. Determination of Polymer Additives by Liquid Chromatography Coupled with Mass Spectrometry. A Comparison of Atmospheric Pressure Photoionization (APPI), Atmospheric Pressure Chemical Ionization (APCI), and Electrospray Ionization (ESI). Polym. Degrad. Stab. 2009, 94 (8), 1213–1219. 10.1016/j.polymdegradstab.2009.04.021.

[ref34] Leinonen A.; Kuuranne T.; Kostiainen R. Liquid Chromatography/Mass Spectrometry in Anabolic Steroid Analysis—Optimization and Comparison of Three Ionization Techniques: Electrospray Ionization, Atmospheric Pressure Chemical Ionization and Atmospheric Pressure Photoionization. Journal of Mass Spectrometry 2002, 37 (7), 693–698. 10.1002/jms.328. 12125002

[ref35] Fredenhagen A.; Kühnöl J. Evaluation of the Optimization Space for Atmospheric Pressure Photoionization (APPI) in Comparison with APCI. Journal of Mass Spectrometry 2014, 49 (8), 727–736. 10.1002/jms.3401. 25044900

[ref36] Itoh N.; Aoyagi Y.; Yarita T. Optimization of the Dopant for the Trace Determination of Polycyclic Aromatic Hydrocarbons by Liquid Chromatography/Dopant-Assisted Atmospheric-Pressure Photoionization/Mass Spectrometry. J. Chromatogr A 2006, 1131 (1–2), 285–288. 10.1016/j.chroma.2006.08.091. 16996068

[ref37] Riu A.; Zalko D.; Debrauwer L. Study of Polybrominated Diphenyl Ethers Using Both Positive and Negative Atmospheric Pressure Photoionization and Tandem Mass Spectrometry. Rapid Commun. Mass Spectrom. 2006, 20 (14), 2133–2142. 10.1002/rcm.2557. 16773670

[ref38] Short L. C.; Cai S. S.; Syage J. A. APPI-MS: Effects of Mobile Phases and VUV Lamps on the Detection of PAH Compounds. J. Am. Soc. Mass Spectrom. 2007, 18 (4), 589–599. 10.1016/j.jasms.2006.11.004. 17188507 PMC2709839

[ref39] Brauer J. I.; Beech I. B.; Sunner J. Mass Spectrometric Imaging Using Laser Ablation and Solvent Capture by Aspiration (LASCA). J. Am. Soc. Mass Spectrom. 2015, 26 (9), 1538–1547. 10.1007/s13361-015-1176-0. 26122514

[ref40] Kokesch-Himmelreich J.; Wittek O.; Race A. M.; Rakete S.; Schlicht C.; Busch U.; Römpp A. MALDI Mass Spectrometry Imaging: From Constituents in Fresh Food to Ingredients, Contaminants and Additives in Processed Food. Food Chem. 2022, 385, 132529 10.1016/j.foodchem.2022.132529. 35279497

[ref41] Martín-Saiz L.; Mosteiro L.; Solano-Iturri J. D.; Rueda Y.; Martín-Allende J.; Imaz I.; Olano I.; Ochoa B.; Fresnedo O.; Fernández J. A.; Larrinaga G. High-Resolution Human Kidney Molecular Histology by Imaging Mass Spectrometry of Lipids. Anal. Chem. 2021, 93 (27), 9364–9372. 10.1021/acs.analchem.1c00649. 34192457 PMC8922278

[ref42] Nizioł J.; Sunner J.; Beech I.; Ossoliński K.; Ossolińska A.; Ossoliński T.; Płaza A.; Ruman T. Localization of Metabolites of Human Kidney Tissue with Infrared Laser-Based Selected Reaction Monitoring Mass Spectrometry Imaging and Silver-109 Nanoparticle-Based Surface Assisted Laser Desorption/Ionization Mass Spectrometry Imaging. Anal. Chem. 2020, 92 (6), 4251–4258. 10.1021/acs.analchem.9b04580. 32083846 PMC7497619

[ref43] Kokesch-Himmelreich J.; Wittek O.; Race A. M.; Rakete S.; Schlicht C.; Busch U.; Römpp A. MALDI Mass Spectrometry Imaging: From Constituents in Fresh Food to Ingredients, Contaminants and Additives in Processed Food. Food Chem. 2022, 385, 132529 10.1016/j.foodchem.2022.132529. 35279497

[ref44] Razgonova M.; Boyko A.; Zinchenko Y.; Tikhonova N.; Sabitov A.; Mikhailovich Zakharenko A.; Golokhvast K. Actinidia Deliciosa: A High-Resolution Mass Spectrometric Approach for the Comprehensive Characterization of Bioactive Compounds. Turk. J. Agric. For 2023, 155 10.55730/1300-011X.3074.

[ref45] Takeoka G. R.; Güntert M.; Jennings W.; Flath R. A.; Wurz R. E. Volatile Constituents of Kiwi Fruit (Actinidia Chinensis Planch.). J. Agric. Food Chem. 1986, 34 (3), 576–578. 10.1021/jf00069a050.

[ref46] Skypala I. J.; Reese I. Managing Individuals with Non-Immune Food Hypersensitivity. Ref. Module Food Sci. 2023, 10.1016/B978-0-323-96018-2.00129-2.

[ref47] Gondor O. K.; Pál M.; Janda T.; Szalai G. The Role of Methyl Salicylate in Plant Growth under Stress Conditions. J. Plant Physiol 2022, 277, 153809 10.1016/j.jplph.2022.153809. 36099699

[ref48] Lewis D. H.; Burge G. K.; Schmierer D. M.; Jameson P. E. Cytokinins and Fruit Development in the Kiwifruit (Actinidia Deliciosa). I. Changes during Fruit Development. Physiol. Plant 1996, 98 (1), 179–186. 10.1034/j.1399-3054.1996.980122.x.

[ref49] Álvarez T.; Ramírez R. The Metabolism of Oxalic Acid. Turk. J. Zool. 2000, 24 (1), 103–106.

[ref50] Li P.; Liu C.; Luo Y.; Shi H.; Li Q.; Pinchu C.; Li X.; Yang J.; Fan W. Oxalate in Plants: Metabolism, Function, Regulation, and Application. J. Agric. Food Chem. 2022, 70 (51), 16037–16049. 10.1021/acs.jafc.2c04787. 36511327

[ref51] Lee H. S.; Kim E. N.; Jeong G. S. Ameliorative Effect of Citropten Isolated from Citrus Peel Extract as a Modulator of T Cell and Intestinal Epithelial Cell Activity in DSS-Induced Colitis. Molecules 2022, 27 (14), 4633 10.3390/molecules27144633. 35889507 PMC9321940

[ref52] Sun Y. L.; Hong S. K. Effects of Plant Growth Regulators and L-Glutamic Acid on Shoot Organogenesis in the Halophyte Leymus Chinensis (Trin.). Plant Cell Tissue Organ Cult 2010, 100 (3), 317–328. 10.1007/s11240-009-9653-4.

[ref53] Alfosea-Simón M.; Simón-Grao S.; Zavala-Gonzalez E. A.; Cámara-Zapata J. M.; Simón I.; Martínez-Nicolás J. J.; Lidón V.; García-Sánchez F. Physiological, Nutritional and Metabolomic Responses of Tomato Plants After the Foliar Application of Amino Acids Aspartic Acid, Glutamic Acid and Alanine. Front Plant Sci. 2021, 11, 581234 10.3389/fpls.2020.581234. 33488641 PMC7817619

[ref54] Seaman C.; Flinders B.; Eijkel G.; Heeren R. M. A.; Bricklebank N.; Clench M. R. afterlife Experiment”: Use of MALDI-MS and SIMS Imaging for the Study of the Nitrogen Cycle within Plants. Anal. Chem. 2014, 86 (20), 10071–10077. 10.1021/ac501191w. 25230319

[ref55] Brauer J. I.; Beech I. B.; Sunner J. Mass Spectrometric Imaging Using Laser Ablation and Solvent Capture by Aspiration (LASCA). J. Am. Soc. Mass Spectrom. 2015, 26 (9), 1538–1547. 10.1007/s13361-015-1176-0. 26122514

[ref56] Wang T.; Fu X.; Chen Q.; Patra J. K.; Wang D.; Wang Z.; Gai Z. Arachidonic Acid Metabolism and Kidney Inflammation. International Journal of Molecular Sciences 2019, 20 (15), 3683 10.3390/ijms20153683. 31357612 PMC6695795

[ref57] Schwartzman M. L.; Martasek P.; Rios A. R.; Levere R. D.; Solangi K.; Goodman A. I.; Abraham N. G. Cytochrome P450-Dependent Arachidonic Acid Metabolism in Human Kidney. Kidney Int. 1990, 37 (1), 94–99. 10.1038/ki.1990.13. 2105407

[ref58] Chesney R. W.; Han X.; Patters A. B. Taurine and the Renal System. J. Biomed. Sci. 2010, 17 (SUPPL. 1), 1–10. 10.1186/1423-0127-17-S1-S4. 20804616 PMC2994373

[ref59] Shankar V.; Vijayalakshmi K.; Nolley R.; Sonn G. A.; Kao C.-S.; Zhao H.; Wen R.; Eberlin L. S.; Tibshirani R.; Zare R. N.; Brooks J. D. Distinguishing Renal Cell Carcinoma From Normal Kidney Tissue Using Mass Spectrometry Imaging Combined With Machine Learning. JCO Precision Oncology 2023, 7, e2200668 10.1200/PO.22.00668. 37285559 PMC10309512

[ref60] Han X.; Yue J.; Chesney R. W. Functional TauT Protects against Acute Kidney Injury. Journal of the American Society of Nephrology 2009, 20 (6), 1323–1332. 10.1681/ASN.2008050465. 19423693 PMC2689910

[ref61] Wettersten H. I.; Hakimi A. A.; Morin D.; Bianchi C.; Johnstone M. E.; Donohoe D. R.; Trott J. F.; Aboud O. A.; Stirdivant S.; Neri B.; Wolfert R.; Stewart B.; Perego R.; Hsieh J. J.; Weiss R. H. Grade-Dependent Metabolic Reprogramming in Kidney Cancer Revealed by Combined Proteomics and Metabolomics Analysis. Cancer Res. 2015, 75 (12), 2541–2552. 10.1158/0008-5472.CAN-14-1703. 25952651 PMC4470795

[ref62] Razgonova M.; Boyko A.; Zinchenko Y.; Tikhonova N.; Sabitov A.; Zakharenko A. M.; Golokhvast K. Actinidia Deliciosa: A High-Resolution Mass Spectrometric Approach for the Comprehensive Characterization of Bioactive Compounds. Turk. J. Agric. For. 2023, 47 (2), 155–169. 10.55730/1300-011X.3074.

[ref63] Zhang J.; Li S. Q.; Lin J. Q.; Yu W.; Eberlin L. S. Mass Spectrometry Imaging Enables Discrimination of Renal Oncocytoma from Renal Cell Cancer Subtypes and Normal Kidney Tissues. Cancer Res. 2020, 80 (4), 689–698. 10.1158/0008-5472.CAN-19-2522. 31843980 PMC7024663

[ref64] Vijayalakshmi K.; Shankar V.; Bain R. M.; Nolley R.; Sonn G. A.; Kao C. S.; Zhao H.; Tibshirani R.; Zare R. N.; Brooks J. D. Identification of Diagnostic Metabolic Signatures in Clear Cell Renal Cell Carcinoma Using Mass Spectrometry Imaging. Int. J. Cancer 2020, 147 (1), 256–265. 10.1002/ijc.32843. 31863456 PMC8571954

[ref65] Jones E. E.; Powers T. W.; Neely B. A.; Cazares L. H.; Troyer D. A.; Parker A. S.; Drake R. R. MALDI Imaging Mass Spectrometry Profiling of Proteins and Lipids in Clear Cell Renal Cell Carcinoma. Proteomics 2014, 14 (7–8), 924–935. 10.1002/pmic.201300434. 24497498 PMC4331029

[ref66] Nizioł J.; Ossoliński K.; Ossoliński T.; Ossolińska A.; Bonifay V.; Sekuła J.; Dobrowolski Z.; Sunner J.; Beech I.; Ruman T. Surface-Transfer Mass Spectrometry Imaging of Renal Tissue on Gold Nanoparticle Enhanced Target. Anal. Chem. 2016, 88 (14), 7365–7371. 10.1021/acs.analchem.6b01859. 27329270

