# Supplementary material for: Direct Three-Dimensional Mass Spectrometry Imaging with Laser Ablation Remote Atmospheric Pressure Photoionization/Chemical Ionization
Source: Anal Chem. 2024 Jul 30;96(32):13326–34. doi: 10.1021/acs.analchem.4c03402 (PMC11325297; doi:10.1021/acs.analchem.4c03402)
Supplement: Supplementary file 1 — ac4c03402_si_001.pdf [file ac4c03402_si_001.pdf]

Supporting Information

Direct three-dimensional mass spectrometry imaging with laser ablation remote atmospheric pressure photoionization/chemical ionization (LARAPPI/CI)

Tomasz Ruman,[1]* Zuzanna Krupa[2] and Joanna Nizioł[1]

[1]Department of Inorganic and Analytical Chemistry, Faculty of Chemistry, Rzeszów University of Technology, 6 Powstańców Warszawy Ave., 35-959 Rzeszów, Poland.

[2]Doctoral School of Engineering and Technical Sciences at the Rzeszów University of Technology, 8 Powstańców Warszawy Ave., 35-959 Rzeszów, Poland.

Table of Contents

S1 Optical microscope photographs of ablation tests performed on laser printer paper covered twice with ink without diffraction optical element and with element.

S2 Optical microscope photographs of agar gel (with titanium dioxide) as the result of triple ablation in the visible area with the use of LARAPPI/CI MSI system

S3 Optimization results of solvent mixture and dopant of LARAPPI/CI in negative ion mode.

S4 Comparison of APCI and ESI with and without APPI for compounds tested in agar gel: registered spectra in the full *m/z* range

S5 Comparison of APCI and ESI with and without APPI for tested compounds in agar gel: spectra fragments showing test compounds

S6 Results of the UHPLC-HRMS analysis of radish extracts

S7 Results of optimization of ablation resolution

S8 LARAPPI/CI 2D and 3D MSI control and analysis software

S9 Optimization of LARAPPI/CI working conditions

Table S1. Comparison of the efficiency of ionization methods performed by ablation of agar gel containing test compounds and mass spectrometric measurements.

S1. Optical microscope photographs (different magnifications) of ablation tests performed on two-times-covered laser printer paper (Pentel N850 blue marker) without diffraction optical element (left) and with element (right). Shooting time 0.5 s. The size of the ablation craters (measured with the use of a motorized microscope table) at the paper level was 80 (left) and 170x170 (right) µm.

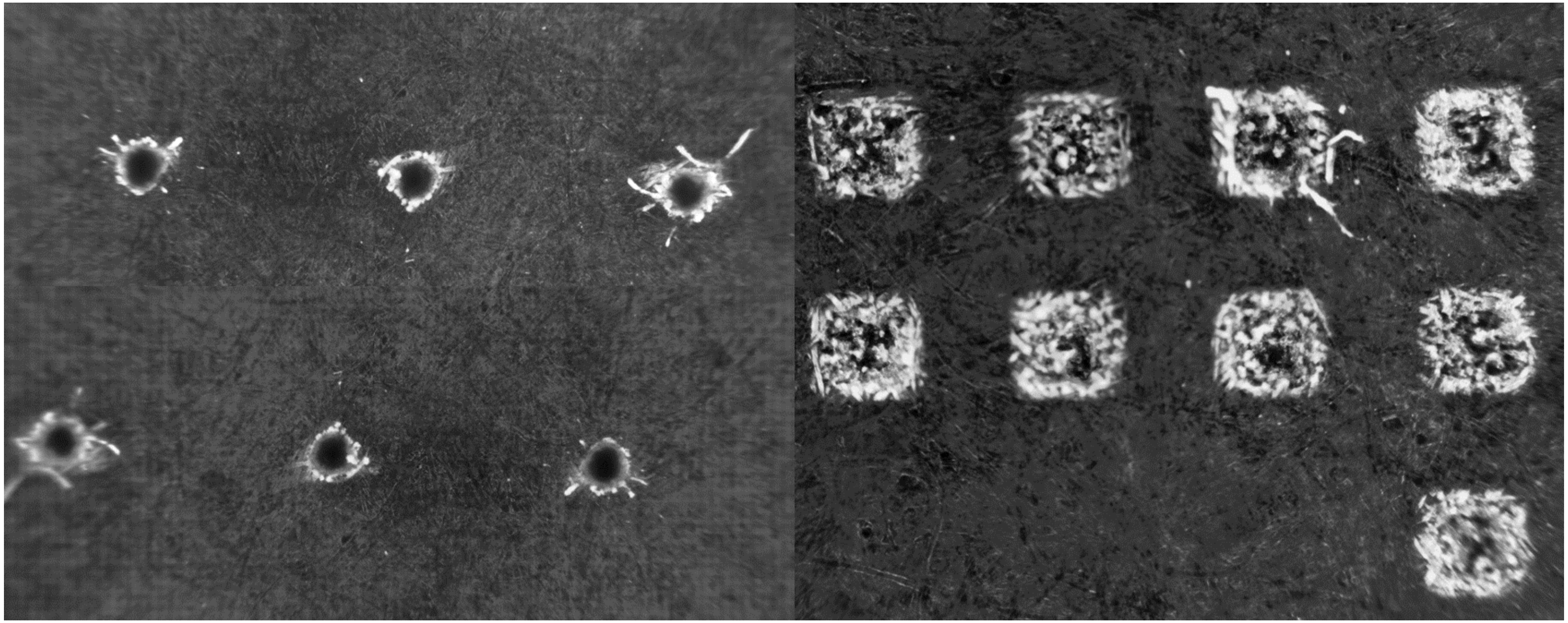

S2. Optical microscope photographs of agar gel (with titanium dioxide) as the result of triple ablation in the visible area with the use of the LARAPPI/CI MSI system. The average depth after ablation of the first layer (measured with a built-in distance sensor) was 0.23 mm,, after the second one 0.47 mm and 0.73 mm after the third. The final depth was also confirmed by measuring with a motorized optical microscope. The photograph on the left has the focus set at the photo on the top level of the gel, the middle is on the walls of the photo on the ablated volume, and the right is on the bottom of the ablated gel. Resolution – 11x7 pixels (X x Y axis), each voxel was ablated for 500 ms (10 laser shots). The dimensions of the ablated region visible below were *ca.* 1.8x1.0x0.73 mm (X x Y x Z, horizontal x vertical x depth).

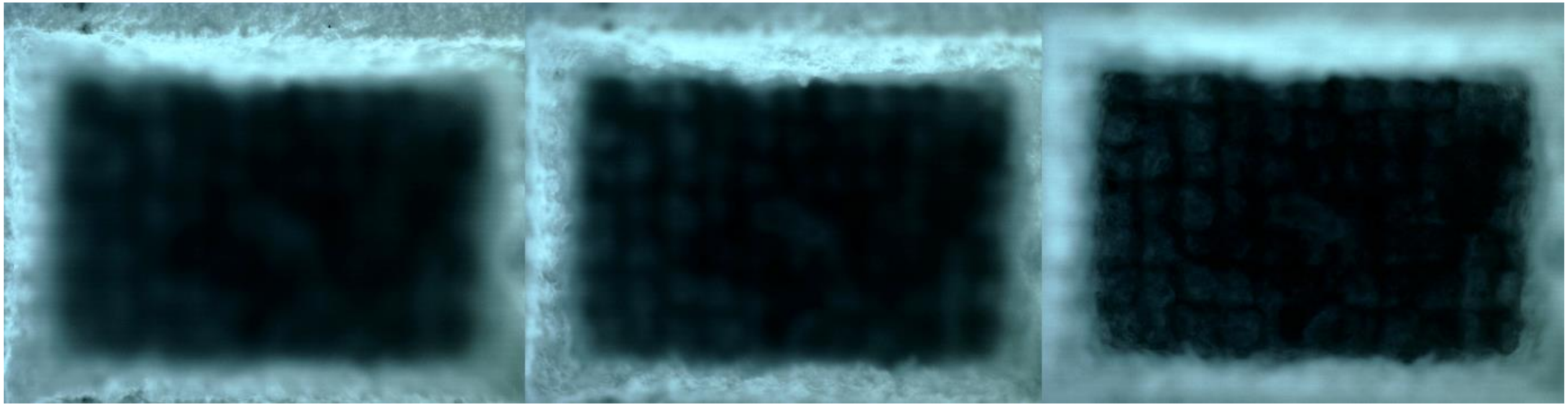

S3. Optimization results of the solvent mixture and dopant of LARAPPI/CI in negative ion mode. The highest signal in the center of the spectrum fragment is [uracil-H]$^-$:

1% toluene in methanol

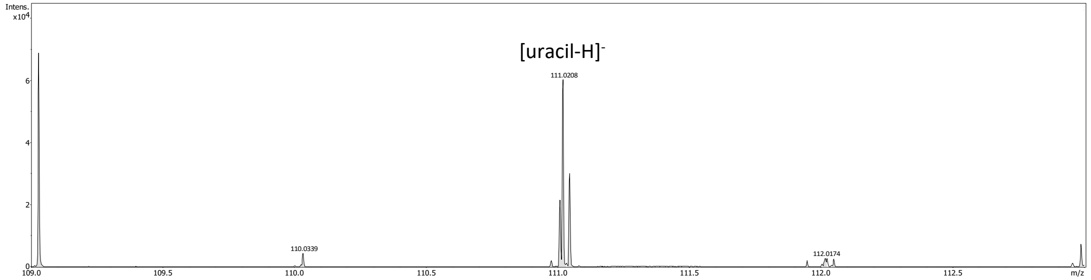

5% toluene in methanol

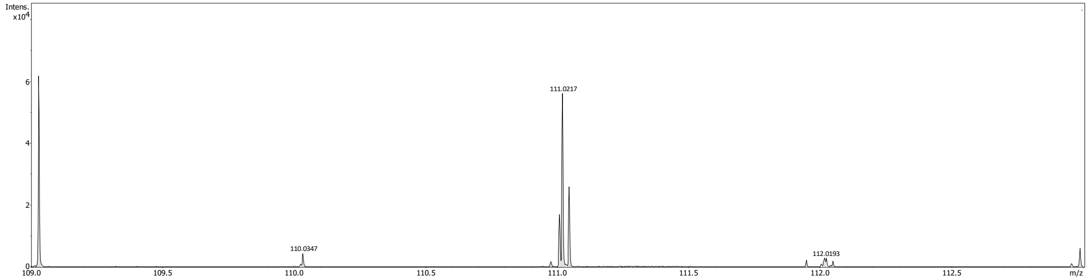

10% toluene in methanol

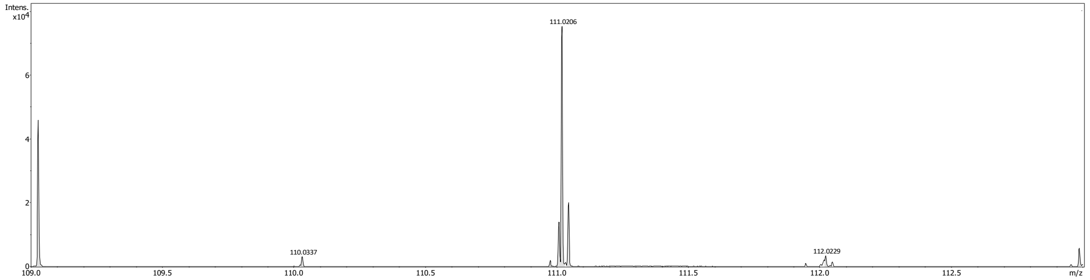

10% toluene and 1% formic acid in methanol

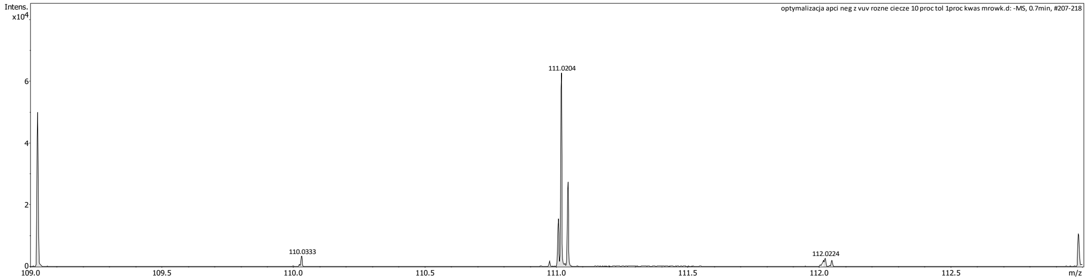

Optimization results of the solvent mixture and dopant of LARAPPI/CI in negative ion mode. The highest signal in the center of the spectrum fragment is [ribose-H]$^-$:

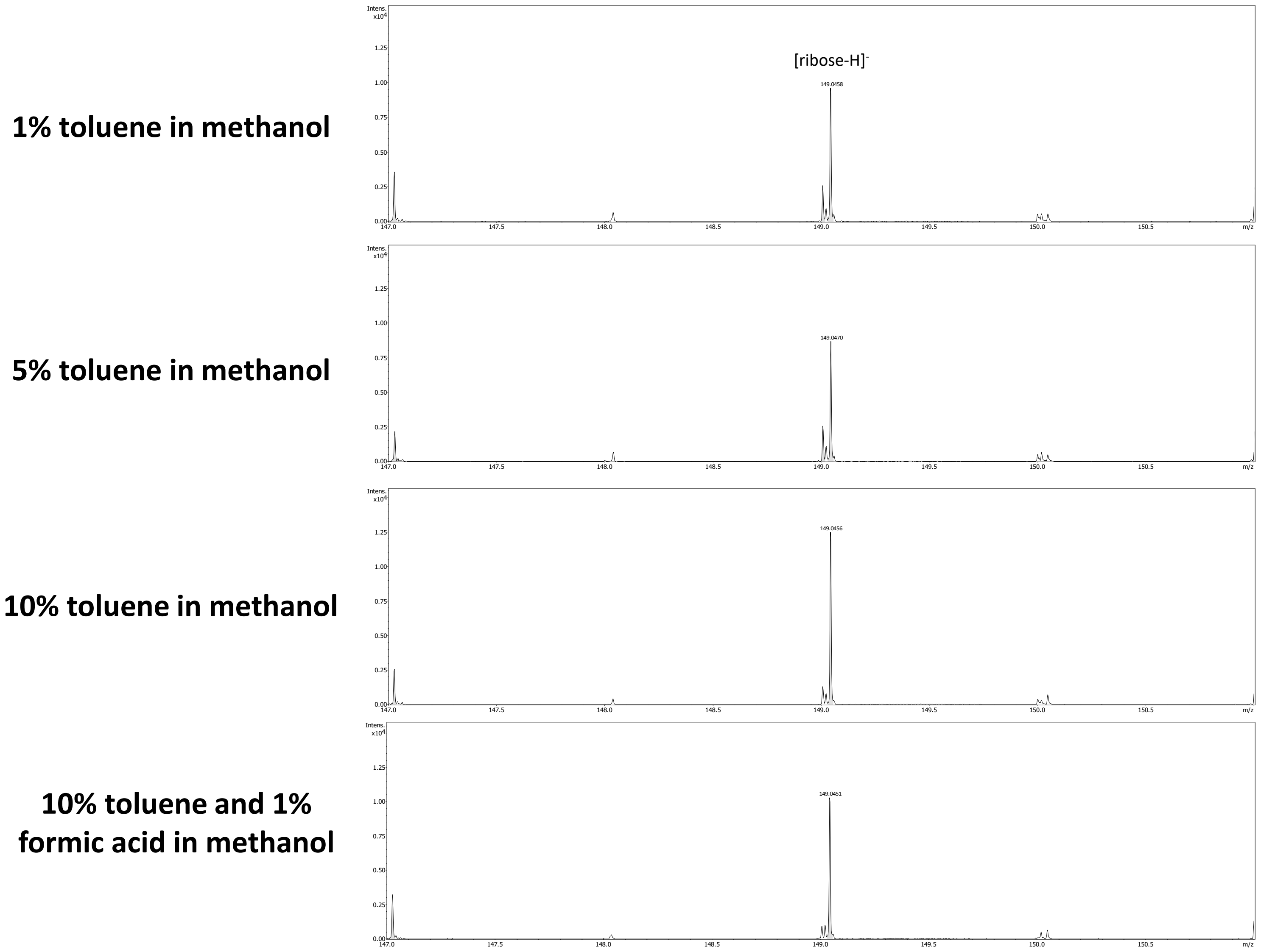

Optimization results of the solvent mixture and dopant of LARAPPI/CI in negative ion mode. The highest signal in the center of the spectrum fragment is [histidine-H]$^-$:

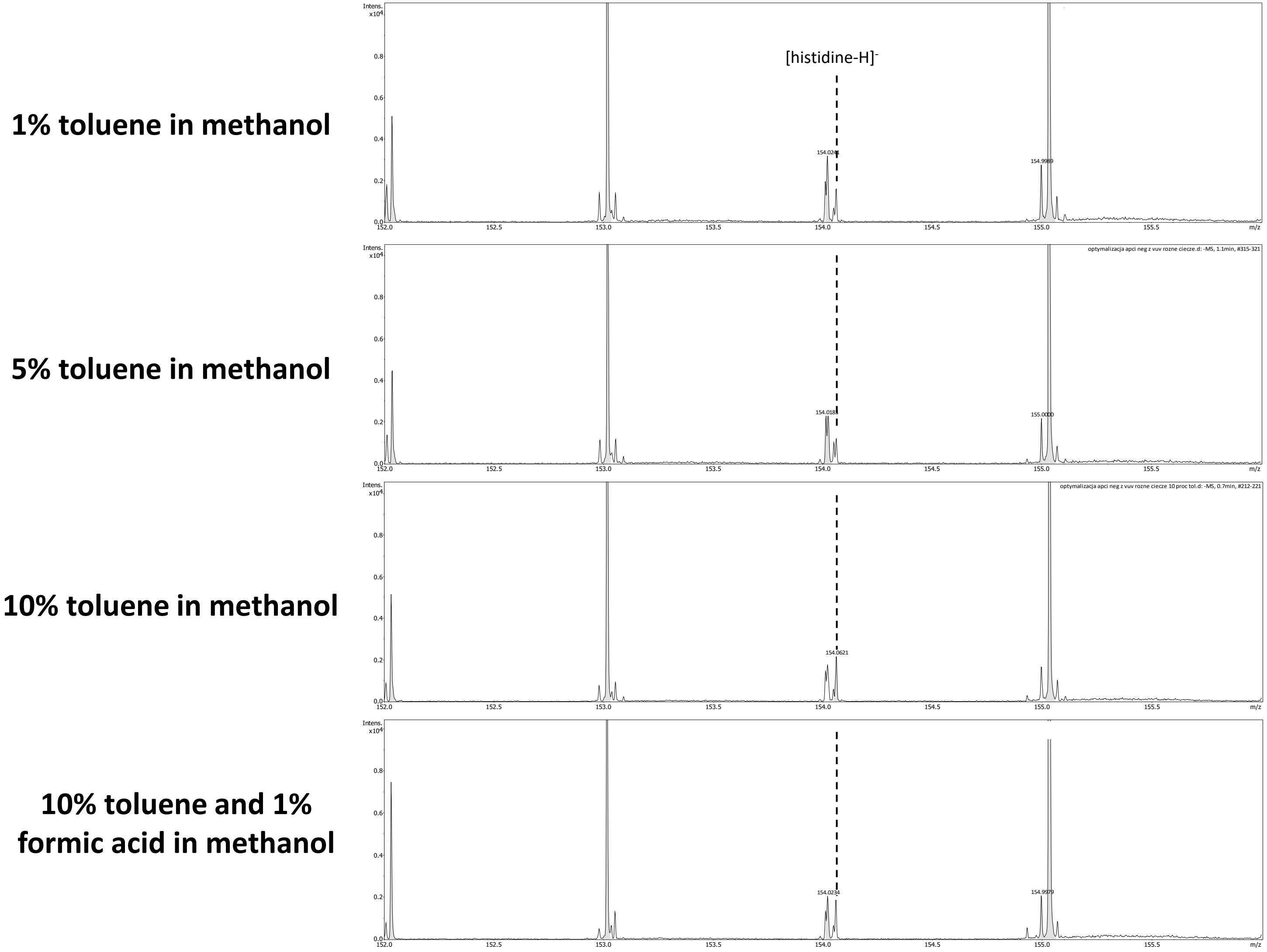

Optimization results of the solvent mixture and dopant of LARAPPI/CI in negative ion mode. The highest signal in the center of the spectrum fragment is [thymidine-H]$^{-}$:

1% toluene in methanol

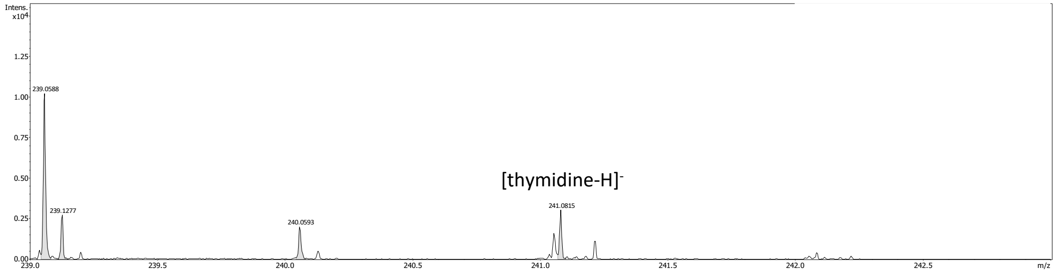

5% toluene in methanol

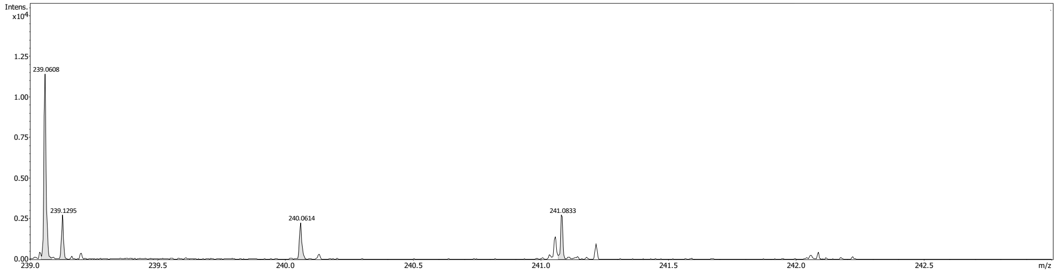

10% toluene in methanol

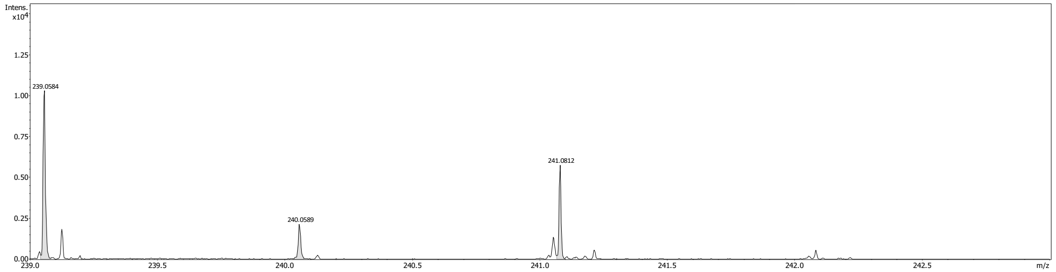

10% toluene and 1% formic acid in methanol

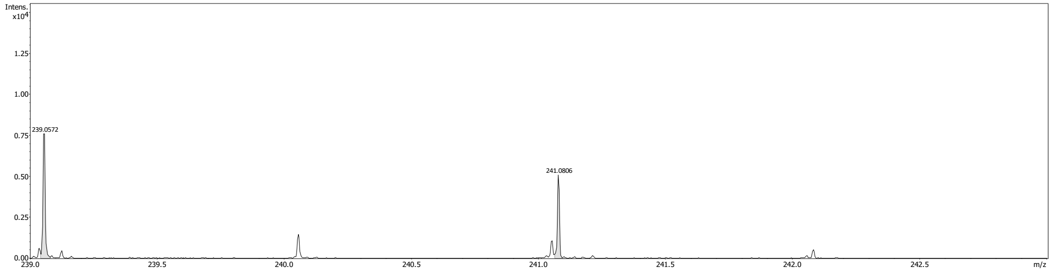

S4. Comparison of APCI and ESI with and without APPI for compounds tested on agar gel: registered spectra in the full *m/z* range. The vertical scale is identical in all six spectra.

APCI positive mode

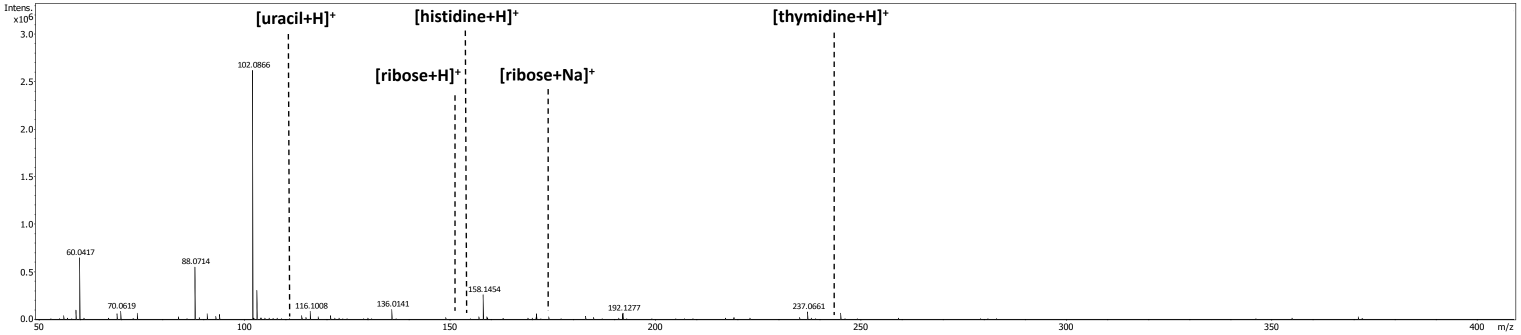

APCI/APPI positive mode

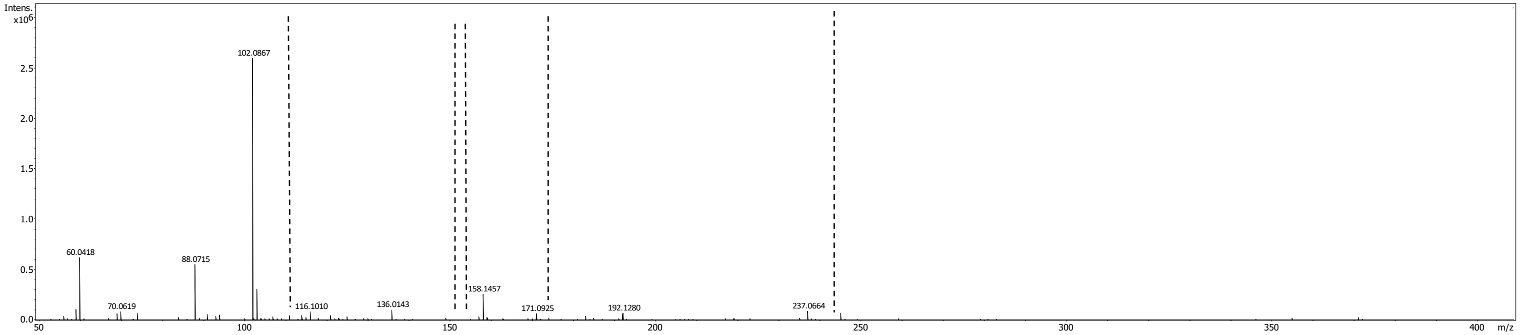

ESI positive mode

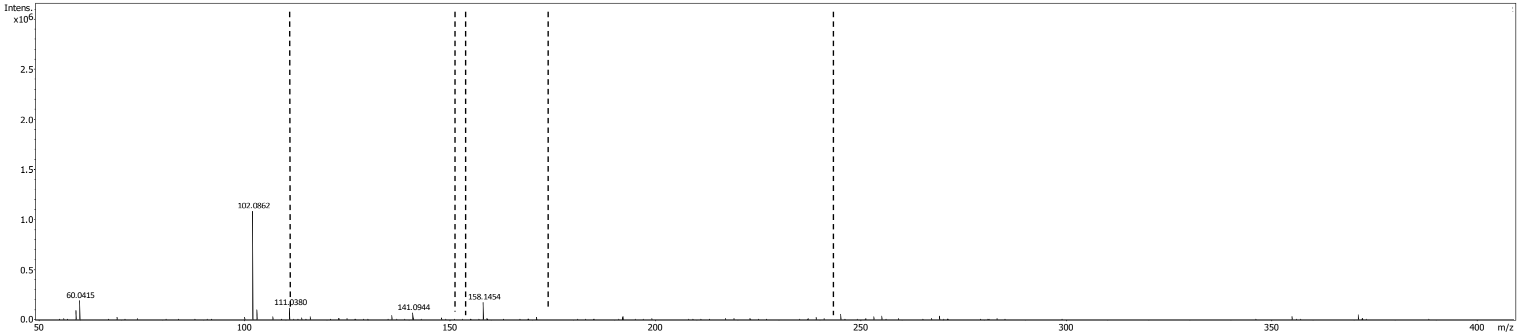

APCI negative mode

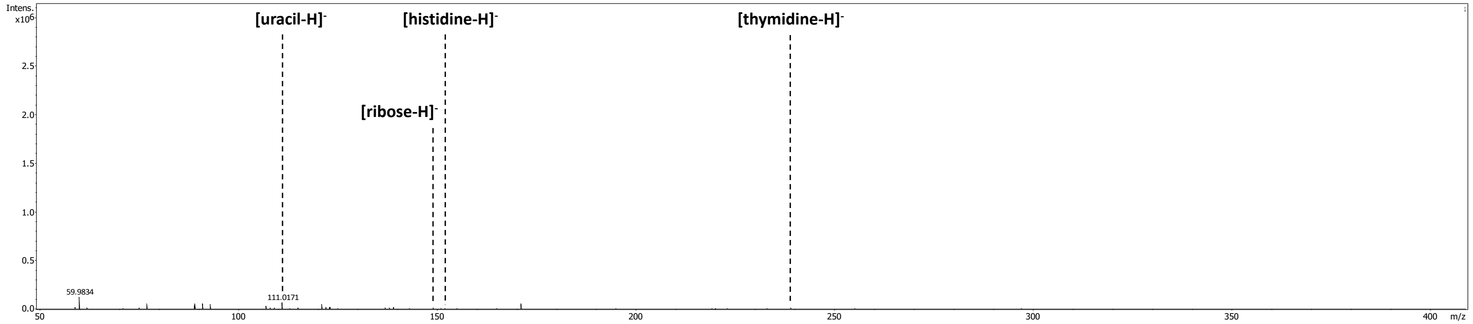

APCI/APPI negative mode

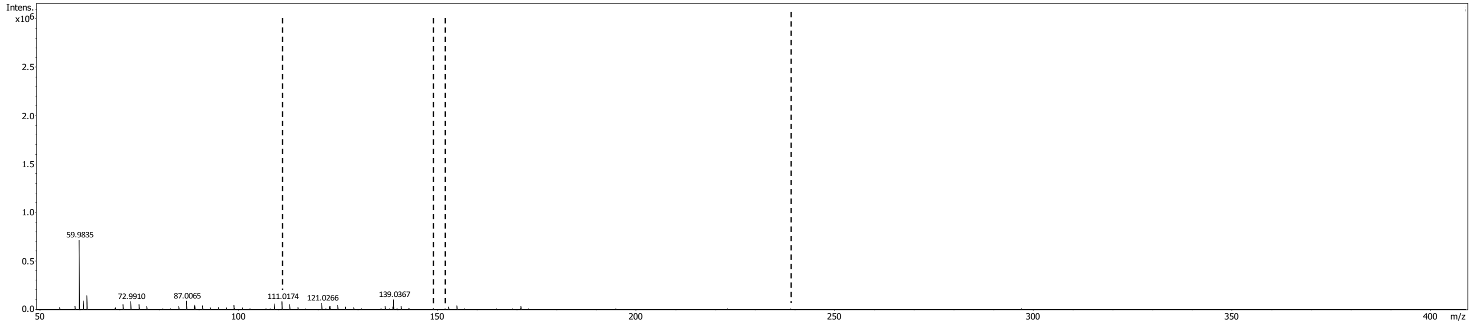

ESI negative mode

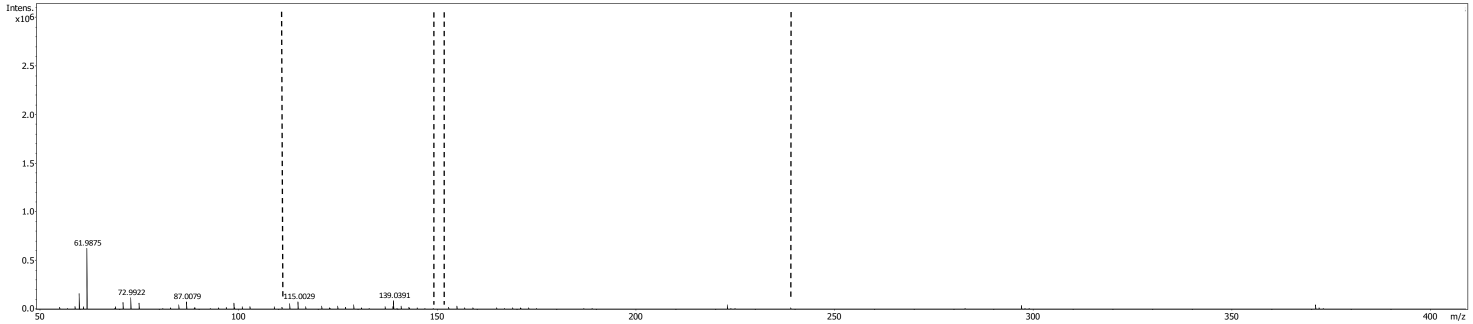

S5. Comparison of APCI and ESI with and without APPI for tested compounds in agar gel: spectra fragments showing test compounds

Histidine positive mode

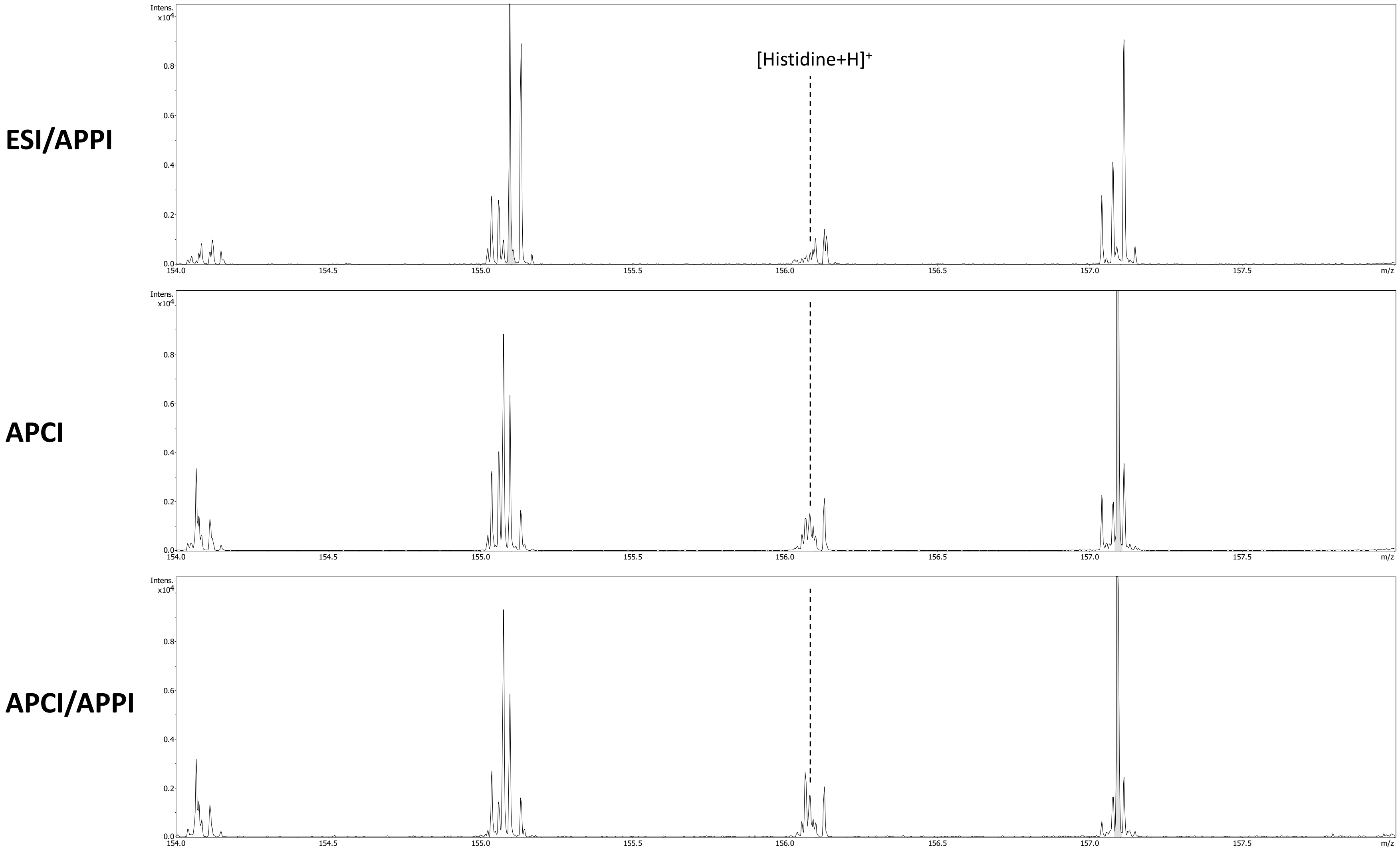

Ribose positive mode

ESI/APPI

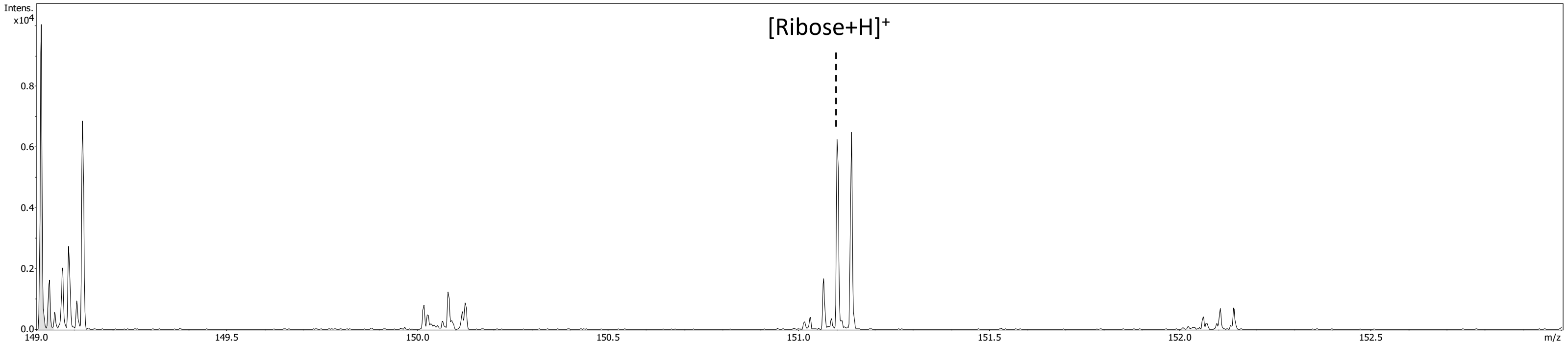

APCI

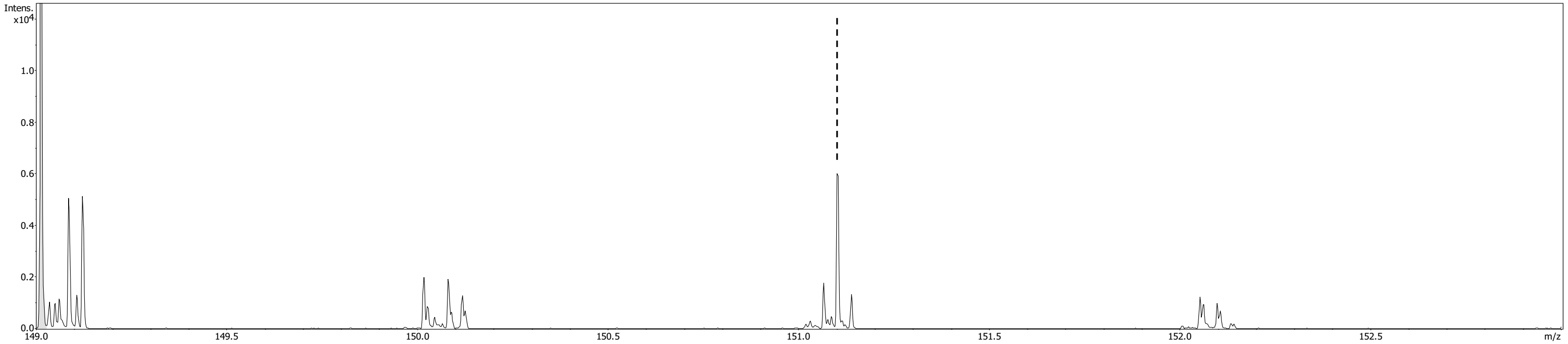

APCI/APPI

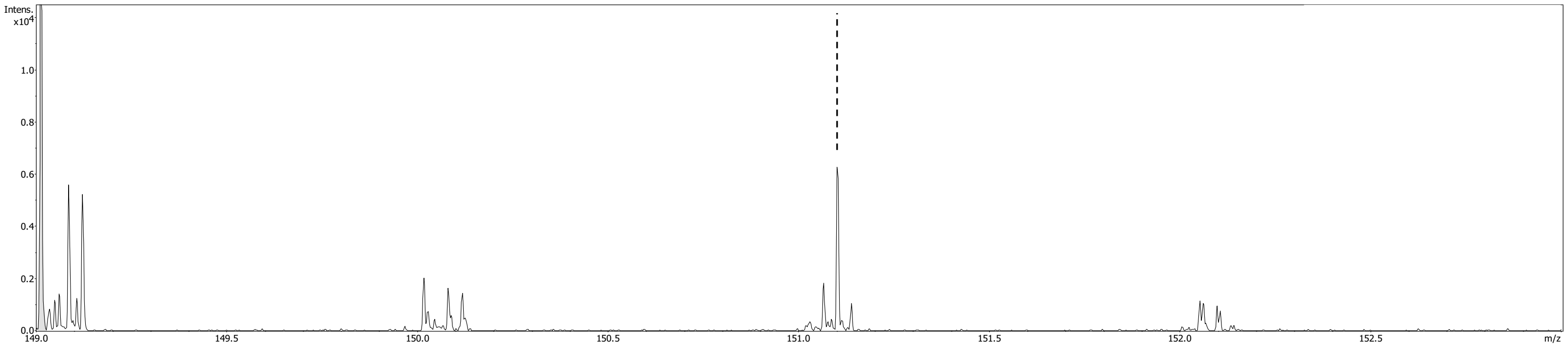

Uracil positive mode

ESI/APPI

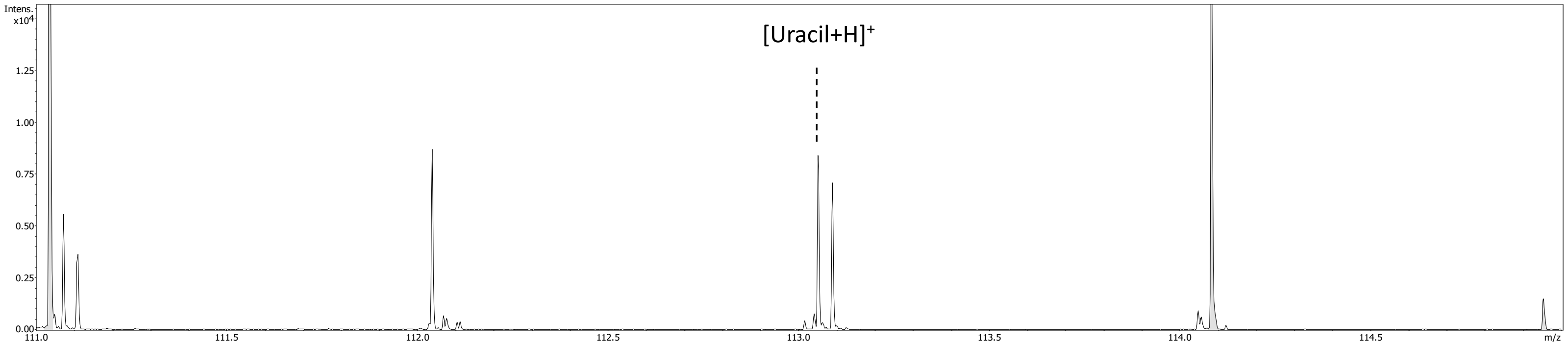

APCI

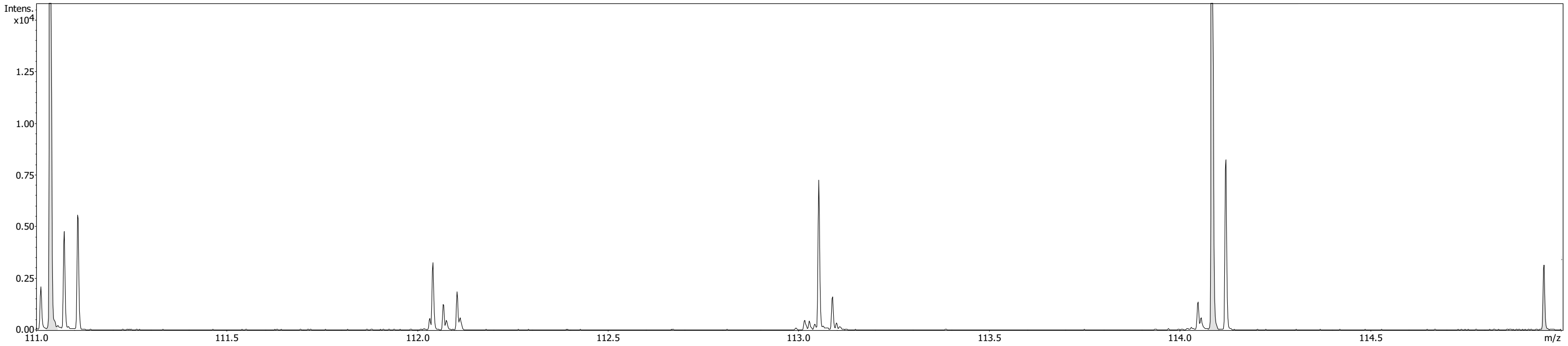

APCI/APPI

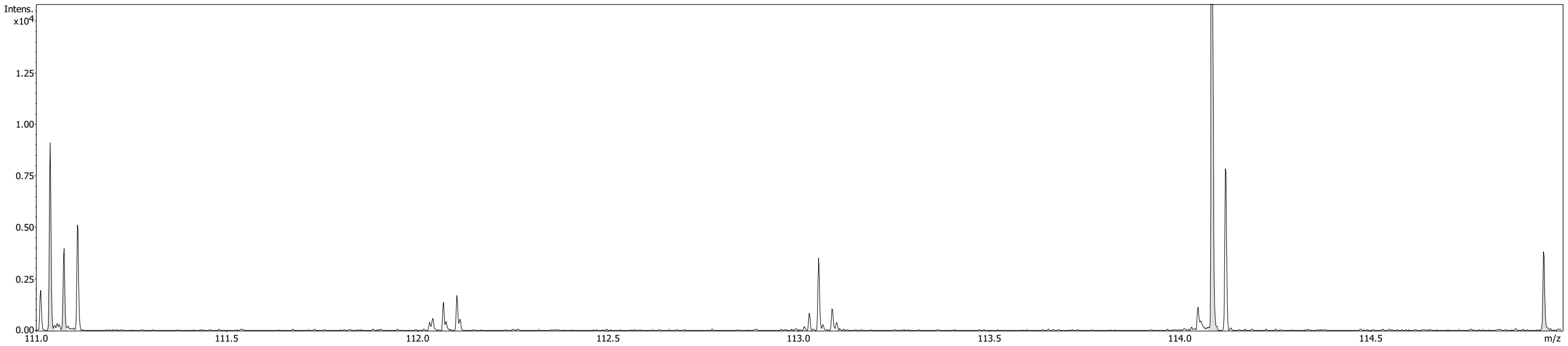

Thymidine positive mode

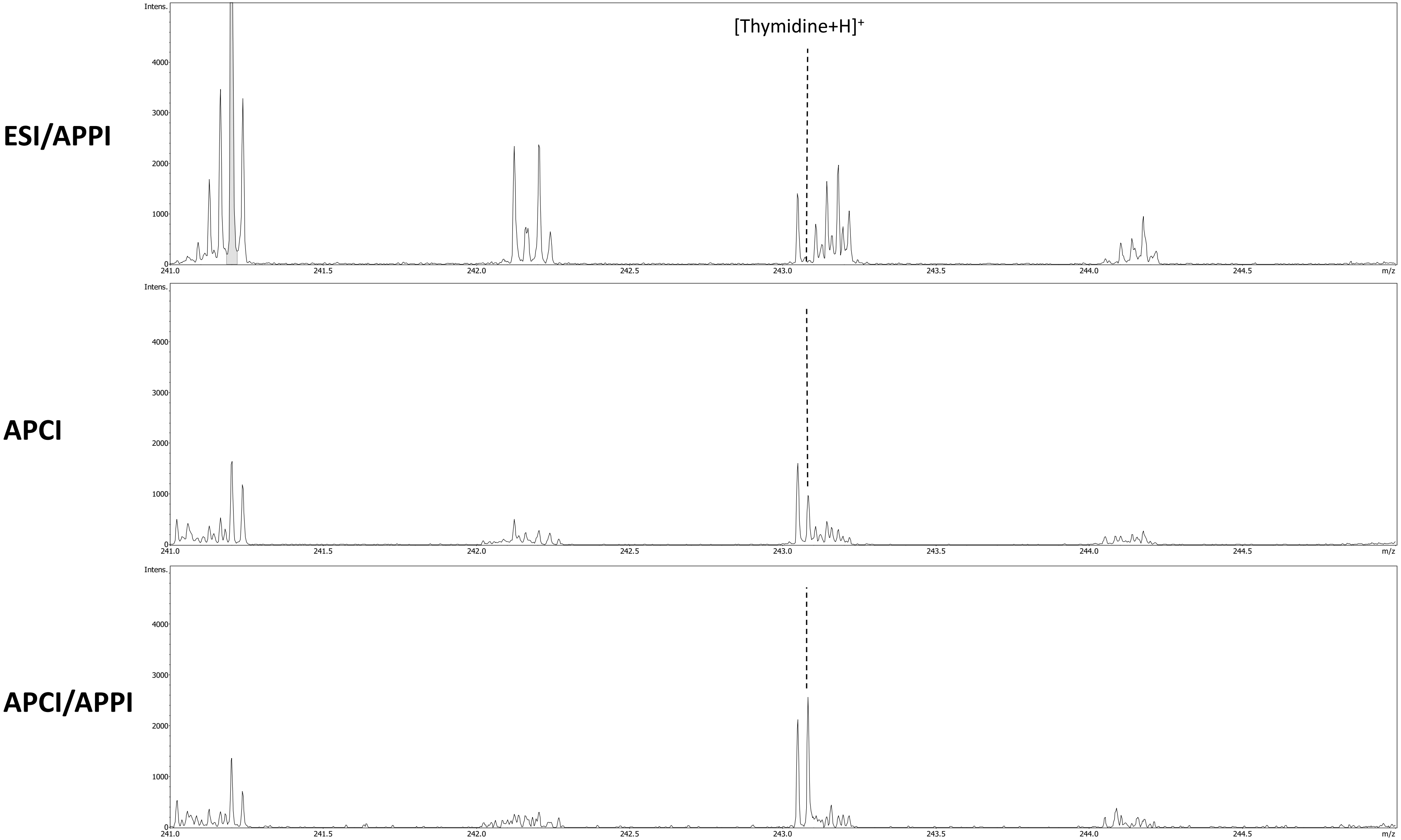

Thymidine negative mode

ESI/APPI

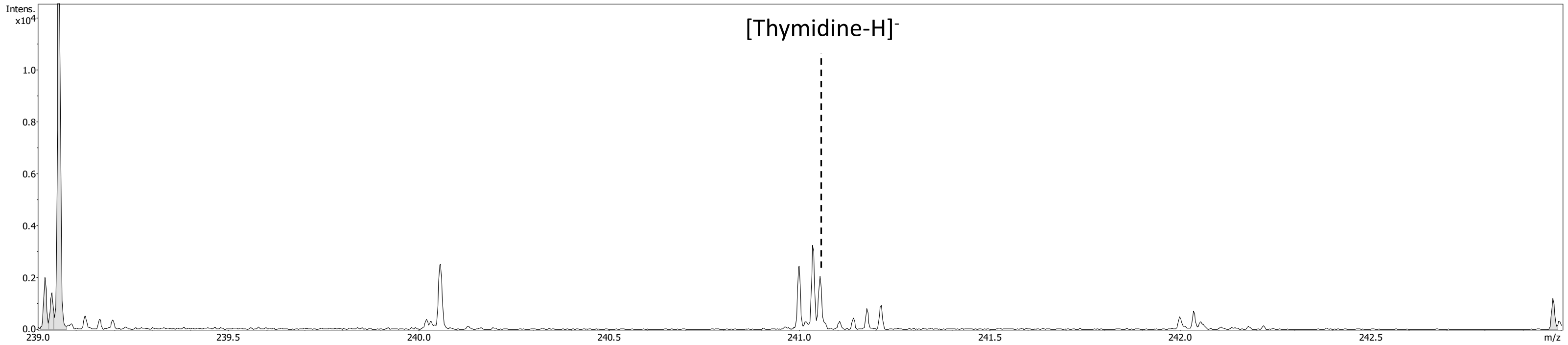

APCI

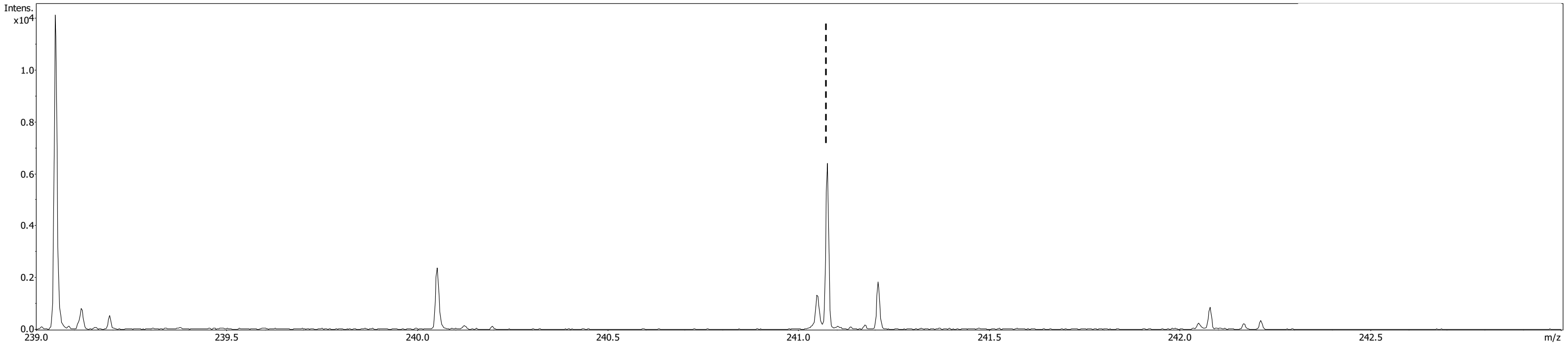

APCI/APPI

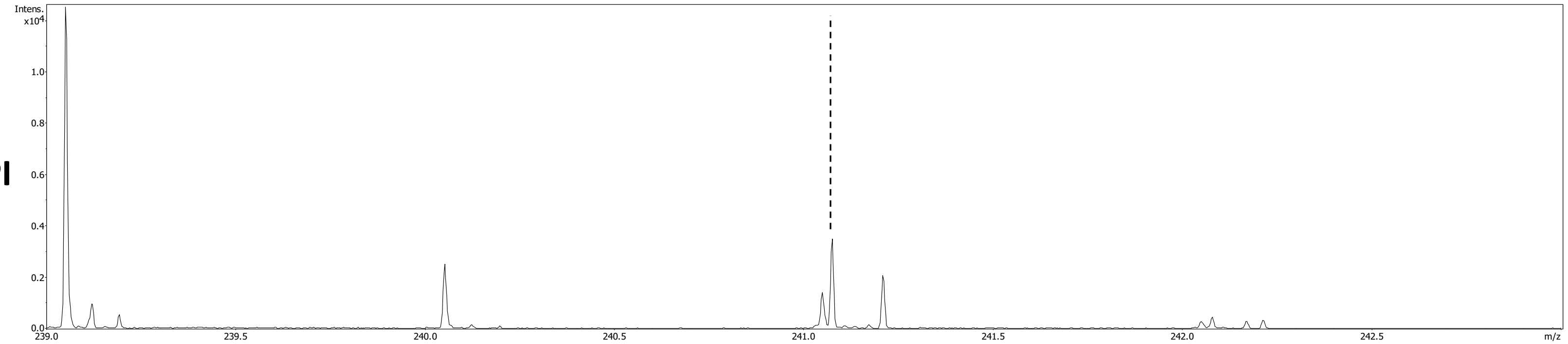

Ribose negative mode

ESI/APPI

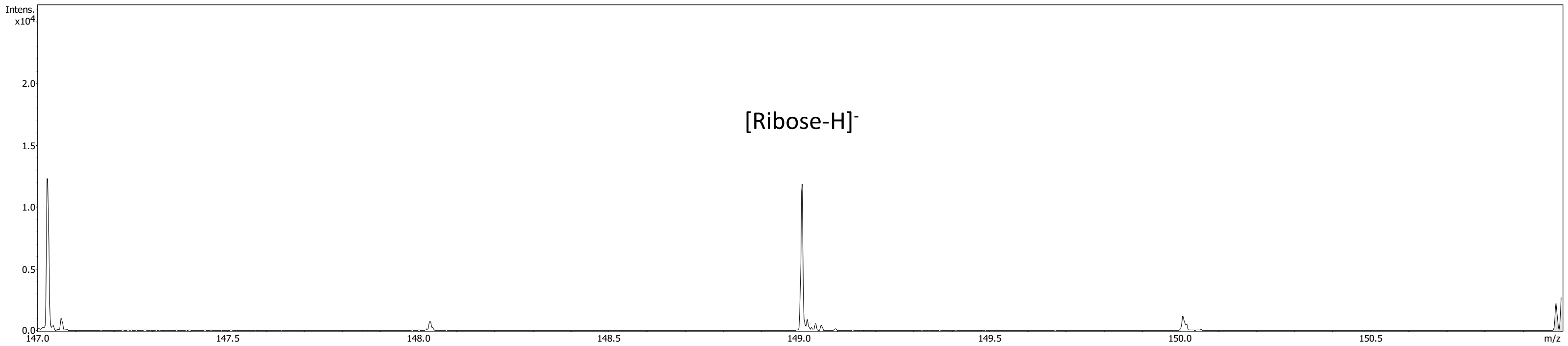

APCI

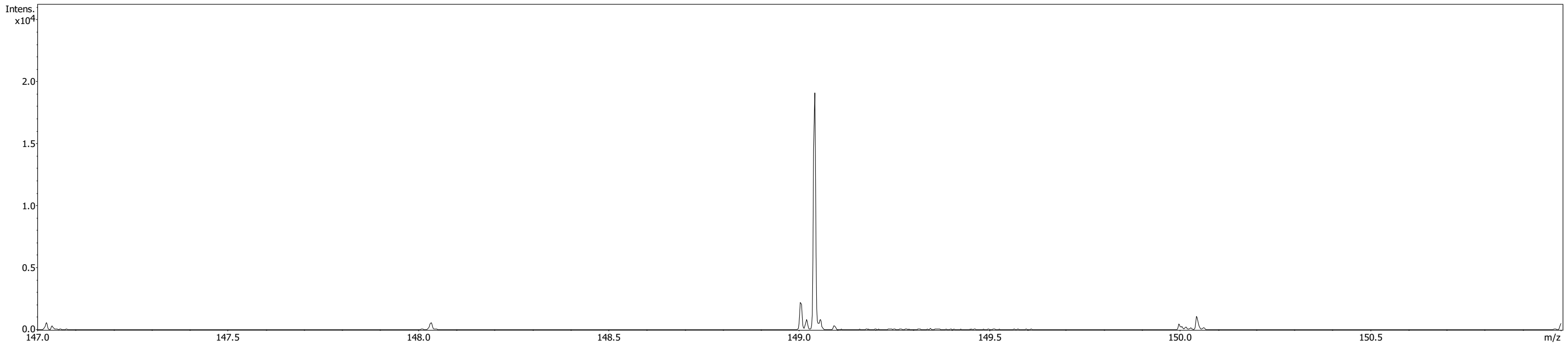

APCI/APPI

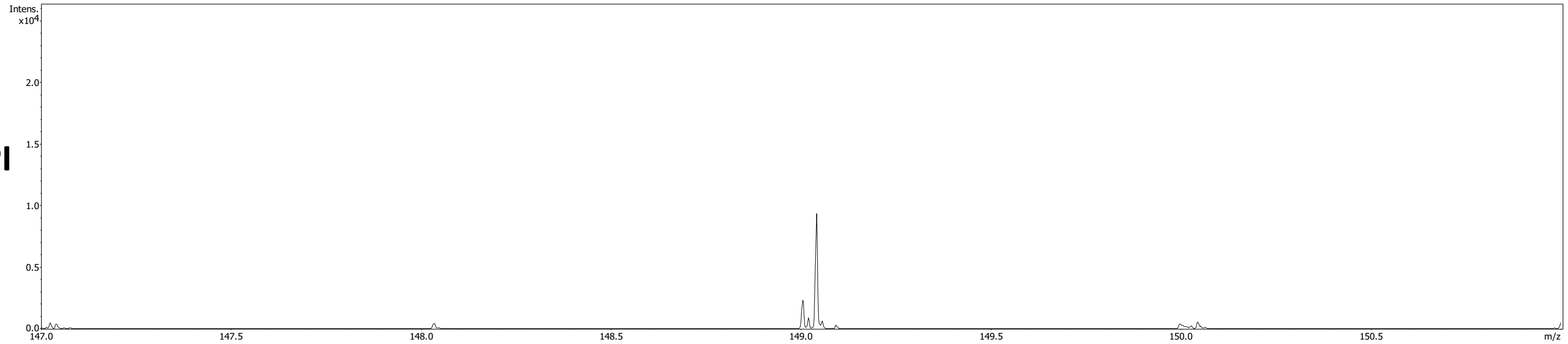

Uracil negative mode

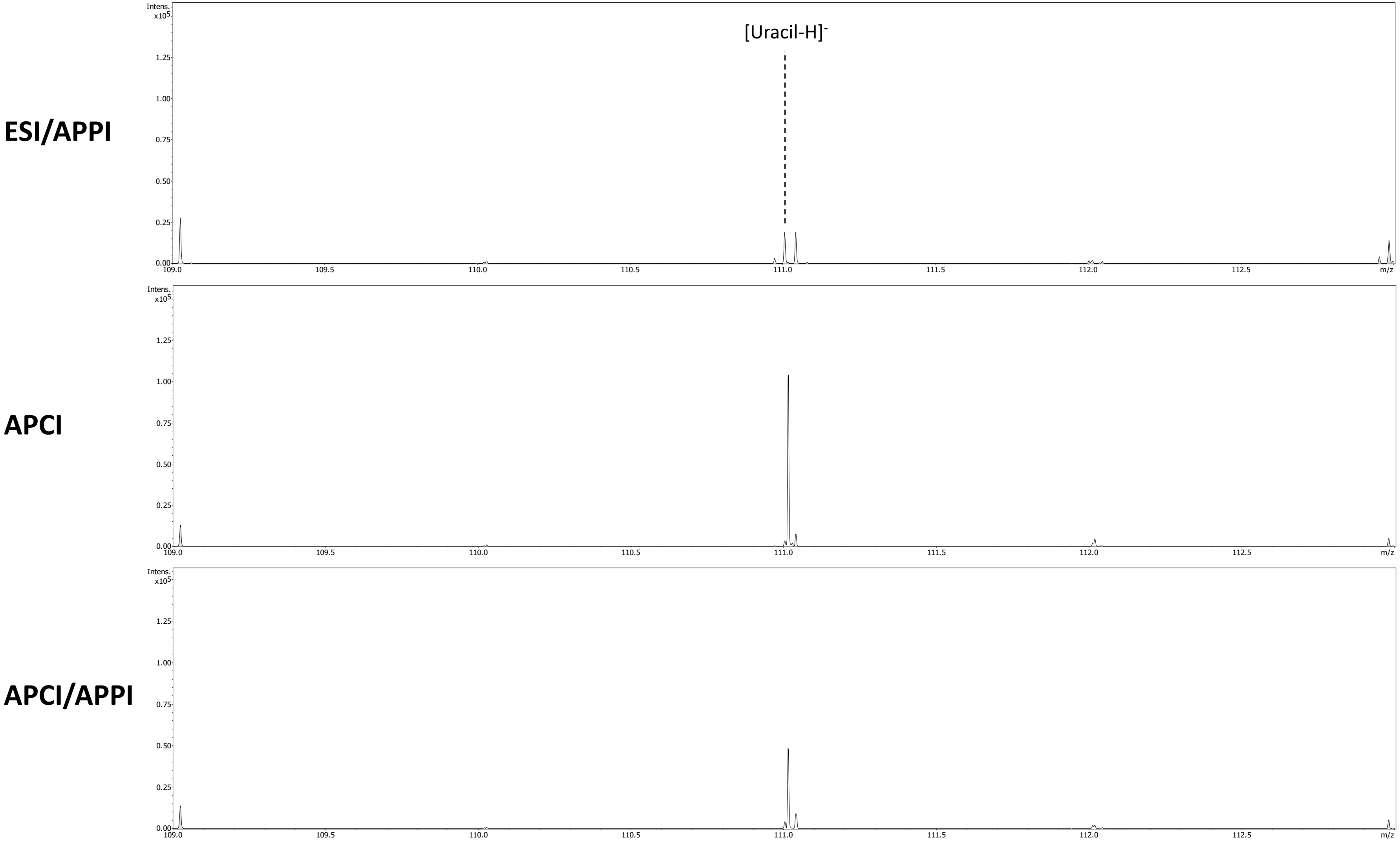

Histidine negative mode

ESI/APPI

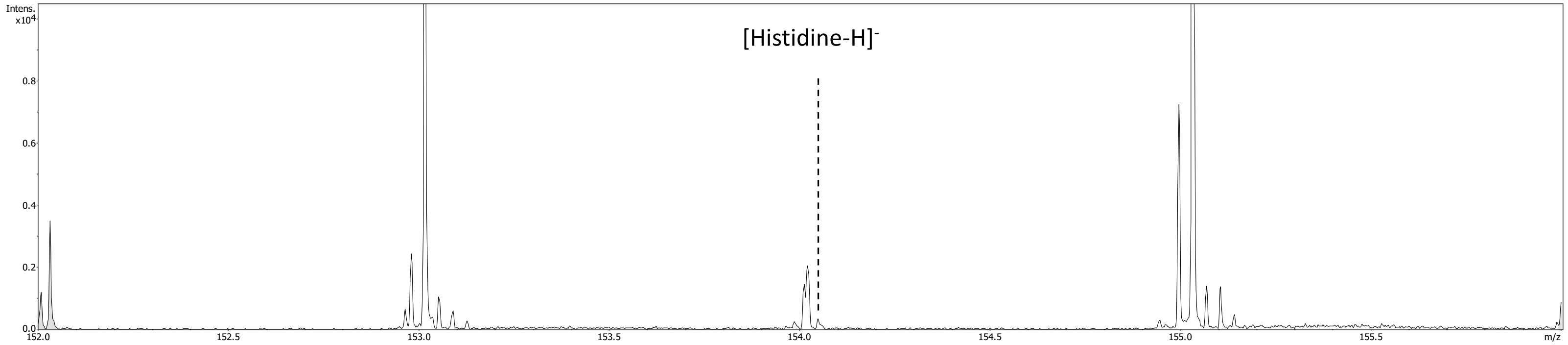

APCI

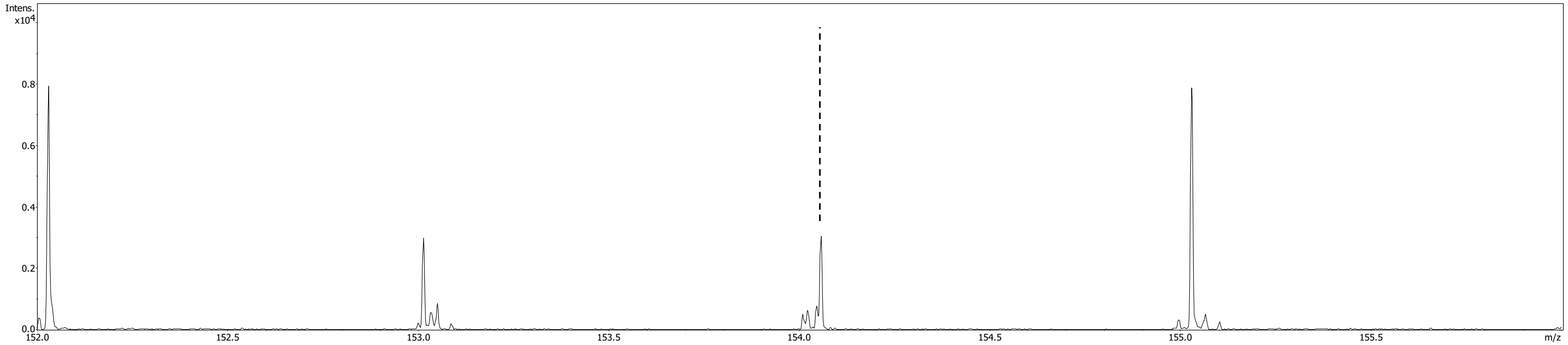

APCI/APPI

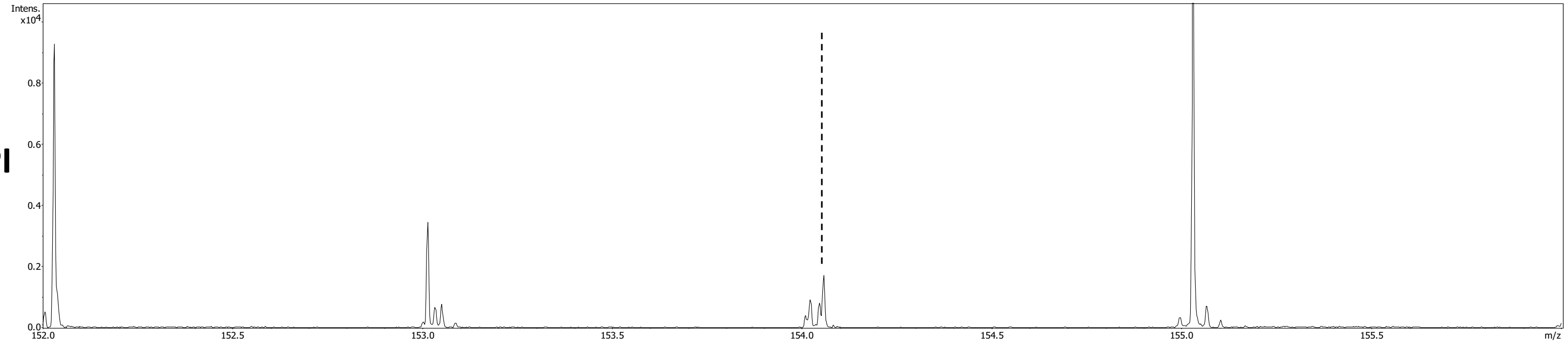

S6. Results of UHPLC-HRMS analysis of radish extracts

Name	Molecular formula	Ion polarity	Formula of detected ions	RT [s]	*m/z*meas.[a]	Mmeas.[b]	Δ*m/z* [ppm]	ΔRT	MS/MS score	Mean abundance
(2-Methyl-6,7-dihydro-1H-[1,4]dioxino[2,3-f]benzimidazol-1-yl)acetic acid	$C_{12}H_{12}N_2O_4$	NEG	$[M-H]^-$	116.5	247.0722	248.0795	-0.8	-	643.9	8846
Linolenic acid	$C_{18}H_{30}O_2$	POS	$[M+H]^+$, $[M+Na]^+$	293.7	279.2315	278.2243	-1.3	-	907.3	47912
[(2R,3S,4S,5R,6R)-6-[(2S,3S,4S,5R)-3,4-Dihydroxy-2,5-bis(hydroxymethyl)oxolan-2-yl]oxy-3,4,5-trihydroxyoxan-2-yl]methyl (E)-3-(4-hydroxy-3,5-dimethoxyphenyl)prop-2-enoate	$C_{23}H_{32}O_{15}$	NEG	$[M-H]^-$	118.6	547.1660	548.1733	-1.5	-	784.7	5516
1-(4-Hydroxyphenyl)-3-[(2R,3R,4S,5S,6R)-3,4,5-trihydroxy-6-(hydroxymethyl)oxan-2-yl]oxypropan-1-one	$C_{15}H_{20}O_8$	NEG	[M-H]-	113.7	327.1082	328.1155	-1.0	-	911.4	3186
12-Cytisineacetamide	$C_{13}H_{17}N_3O_2$	POS	$[M+H]^+$	124.7	248.1387	247.1314	-2.6	-	608.8	12112
13-Keto-9Z,11E-octadecadienoic acid	$C_{18}H_{30}O_3$	POS	$[M+H]^+$, $[M+H-H_2O]^+$	187.1	295.2263	294.2192	-1.6	-	693.9	3516
1-Monolinolenin	$C_{21}H_{36}O_4$	POS	$[M+H]^+$, $[M+Na]^+$, $[M+H-H_2O]^+$	278.0	353.2678	352.2606	-2.5	-	963.1	61646
1-Monostearin	$C_{21}H_{42}O_4$	POS	$[M+H]^+$, $[M+H-H_2O]^+$, $[M+Na]^+$	327.1	359.3152	358.3079	-1.2	-	633.6	23358
1-Oleoyl-sn-glycero-3-phosphocholine	$C_{26}H_{52}NO_7P$	POS	$[M+H]^+$, $[M+Na]^+$, $[M+K]^+$	266.1	522.3543	521.3473	-2.1	-	940.4	78204
1-Oleoyl-sn-glycero-3-phosphoethanolamine	$C_{23}H_{46}NO_7P$	POS	$[M+H]^+$, $[M+H-H_2O]^+$, $[M+Na]^+$	265.5	480.3074	479.2998	-2.2	-	871.5	62306
1-Palmitoyl-2-azelaoylphosphatidylcholine	$C_{33}H_{64}NO_{10}P$	POS	$[M+H]^+$, $[M+Na]^+$	280.7	666.4341	665.4273	0.1	-	989.3	6754
1-Palmitoylglycerol	$C_{19}H_{38}O_4$	POS	$[M+H-H_2O]^+$, $[M+K]^+$, $[M+NH_4]^+$, $[M+H]^+$, $[M+Na]^+$	309.3	313.2733	330.2768	-1.5	-	978.4	97184

1-Stearoyl-sn-glycero-3-phosphocholine	$C_{26}H_{54}NO_7P$	POS	$[M+H]^+$, $[M+Na]^+$	288.0	524.3701	523.3629	-1.8	-	958.1	35536
2-(4-Hydroxyphenyl)-2-oxoacetic acid	$C_8H_6O_4$	NEG	$[M-H]^-$	123.5	165.0194	166.0267	0.5	-	719.3	4104
2,2'-(Tetradecylimino)diethanol	$C_{18}H_{39}NO_2$	POS	$[M+H]^+$	227.2	302.3046	301.2973	-2.7	-	919.6	9320
2-acetoxy-4-pentadecylbenzoic acid	$C_{24}H_{38}O_4$	POS	$[M+Na]^+$, $[M+H]^+$	331.8	413.2654	390.2762	-2.1	-	606.6	15356
2-Isopropylmalic acid	$C_7H_{12}O_5$	POS	$[M+Na]^+$	105.4	199.0576	176.0683	-0.7	2.1	-	1878
2-Ketobutyric acid	$C_4H_6O_3$	NEG	[M+HCOO]-	42.3	147.0302	102.0320	2.1	-13.5	-	1118
2-Methyl-3-ketovaleric acid	$C_6H_{10}O_3$	NEG	$[M+OH]^-$	54.2	147.0665	130.0632	1.5	-	-	1080
2-Oxohexane	$C_6H_{12}O$	POS	$[M+NH_4]^+$	9.1	118.1223	100.0885	-2.9	-		1206
2-Pyrimidinemethanamine	$C_5H_7N_3$	POS	$[M+H]^+$	25.3	110.0710	109.0637	-2.4	-	659.8	4868
3,4-Dihydroxybenzeneacetic acid	$C_8H_8O_4$	POS	$[M+H]^+$	100.6	169.0492	168.0419	-2.3	5.0	-	1536
3-Aminoisobutanoic acid	$C_4H_9NO_2$	POS	$[M+Na]^+$	21.9	126.0528	103.0636	2.2	3.0	-	1006
3-Hexadec-15-en-7-ynyl-4-hydroxy-5-methyloxolan-2-one	$C_{21}H_{34}O_3$	POS	$[M+H]^+$	347.8	335.2579	334.2506	-0.6	-	821.1	12900
3-Hydroxy-4-keto-gamma-carotene	$C_{40}H_{54}O_2$	POS	$[M+H]^+$	338.0	567.4191	566.4118	-1.0	-	785.5	6644
3-Hydroxy-4-methoxycinnamic aicd	$C_{10}H_{10}O_4$	NEG	$[M-H]^-$	143.0	193.0503	194.0575	-2.0	-	778.0	44340
3-Hydroxymethylglutaric acid	$C_6H_{10}O_5$	NEG	$[M-H]^-$	49.8	161.0456	162.0528	0.1	-6.0	926.9	12066
		POS	$[M+Na]^+$	51.6	185.0418	162.0526	-1.5	-4.2	-	1436
3-Indoleacrylic acid	$C_{11}H_9NO_2$	POS	$[M+H]^+$, $[M+NH_4]^+$, $[M+H-H_2O]^+$, $[M+H-CO_2]^+$	109.4	188.0703	187.0628	-1.4		636.8	458750
3-Methyl-2-oxovaleric acid	$C_6H_{10}O_3$	NEG	$[M+HCOO]^-$	99.8	175.0611	130.0629	-0.6	-3.0	-	3962
3-Methyladipic acid	$C_7H_{12}O_4$	POS	$[M+Na]^+$	111.4	183.0631	160.0738	1.5	2.9	-	1362
3-Phosphoglyceric acid	$C_3H_7O_7P$	POS	$[M+K]^+$	10.8	224.9560	185.9929	-0.3	-8.0	-	1500
3-Phosphonopropanoic acid	$C_3H_7O_5P$	NEG	$[M-H]^-$	256.7	152.9960	154.0033	1.2	-	601.3	1614
Tetrahydro-1h-pyrido-34b-indole-carboxylic-acid	$C_{12}H_{12}N_2O_2$	POS	$[M+H]^+$	121.9	217.0967	216.0894	-2.1	-	924.6	129964
4-(Hexopyranosyloxy)-3-methoxybenzoic acid	$C_{14}H_{18}O_9$	NEG	$[M-H]^-$	100.6	329.0874	330.0947	-1.3	-	919.2	5050
4-Deoxytetronic acid	$C_4H_6O_2$	NEG	$[M-H]^-$	46.9	85.0297	86.0370	2.1	0.9	-	1258
4-Hydroxybenzoate	$C_7H_6O_3$	NEG	$[M-H]^-$	125.4	137.0245	138.0318	0.8		996.1	3916
4-Hydroxycyclohexylcarboxylic acid	$C_7H_{12}O_3$	NEG	$[M+OH]^-$	110.0	161.0819	144.0786	-0.5	13.8		1138

4-Methoxyglucobrassicin (4-Methoxy-3-indolylmethyl glucosinolate)	$C_{17}H_{22}N_2O_{10}S_2$	NEG	$[M-H]^-$	124.4	477.0636	478.0709	-1.4	-	871.0	361642
4-Pyridoxate	$C_8H_9NO_4$	NEG	$[M-H]^-$	52.5	182.0461	183.0534	1.1	-	631.1	2738
5,7-Dimethoxy-2H-chromen-2-one	$C_{11}H_{10}O_4$	POS	$[M+H]^+$	142.0	207.0647	206.0574	-2.4	-	670.7	35836
5-Hydroxy-2-(4-hydroxyphenyl)-3-[(2S,3R,4S,5S,6R)-3,4,5-trihydroxy-6-(hydroxymethyl)oxan-2-yl]oxy-7-[(2S,3R,4R,5R,6S)-3,4,5-trihydroxy-6-methyloxan-2-yl]oxychromen-4-one	$C_{27}H_{30}O_{15}$	POS	$[M+H]^+$, $[M+K]^+$, $[M+Na]^+$	130.5	595.1656	594.1586	-0.2	-	993.4	89356
5'-Methylthioadenosine	$C_{11}H_{15}N_5O_3S$	POS	$[M+H]^+$	108.9	298.0968	297.0895	-0.2	14.8	984.2	8056
5-Phenylisoxazol-3-ol	$C_9H_7NO_2$	NEG	$[M-H]^-$	116.5	160.0404	161.0477	0.2	-	672.1	4542
6-Hydroxydopamine	$C_8H_{11}NO_3$	POS	$[M+NH_4]^+$	48.1	187.1073	169.0735	-2.3		-	3816
6-Methyladenine	$C_6H_7N_5$	POS	$[M+H+CH_3CN]^+$	14.6	191.1039	149.0701	-0.5	-7.9	-	1396
8,11,14-Eicosatrienoic acid	$C_{20}H_{34}O_2$	POS	$[M+NH_4]^+$	280.7	324.2889	306.2551	-2.4	-17.8	-	2172
9,10-Dihydrojasmonic acid	$C_{12}H_{20}O_3$	POS	$[M+H-H_2O]^+$, $[M+H]^+$	180.0	195.1375	212.1406	-2.3	-	668.3	15688
9-Oxo-10(E),12(E)-octadecadienoic acid	$C_{18}H_{30}O_3$	POS	$[M+H]^+$, $[M+Na]^+$, $[M+H-H_2O]^+$	263.1	295.2264	294.2190	-1.3	-	896.0	8738
Acetaldehyde	C_2H_4O	NEG	$[M+HCOO]^-$	31.8	89.0243	44.0261	-1.2	-	-	3222
Acetoin	$C_4H_8O_2$	NEG	$[M+HCOO]^-$	52.5	133.0508	88.0526	1.6	-	-	1388
Adenosine	$C_{10}H_{13}N_5O_4$	POS	$[M+H]^+$, $[M+Na]^+$	50.1	268.1037	267.0963	-1.4	-	997.1	119094
Dimethylarginine	$C_8H_{18}N_4O_2$	POS	$[M+H]^+$	34.4	203.1499	202.1426	-2.0	15.3	-	1982
Azelaic acid	$C_9H_{16}O_4$	NEG	$[M-H]^-$	152.6	187.0971	188.1044	-2.4	14.6	764.2	4946
Benzaldehyde	C_7H_6O	POS	$[M+H+CH_3CN]^+$	90.4	148.0755	106.0417	-1.4	-	-	1004
Benzoic acid + 1O, 2MeO, O-Hex	$C_{15}H_{20}O_{10}$	NEG	$[M-H]^-$	106.3	359.0981	360.1054	-0.6	-	690.3	2820
Benzoic acid + 2O, O-Hex	$C_{13}H_{16}O_9$	NEG	$[M-H]^-$	98.9	315.0719	316.0792	-0.9	-	947.0	10328
Biotin	$C_{10}H_{16}N_2O_3S$	POS	$[M+NH_4]^+$	119.4	262.1222	244.0884	0.7	-	-	1452
C17-Sphinganine	$C_{17}H_{37}NO_2$	POS	$[M+H]^+$	213.1	288.2893	287.2820	-1.5	-	885.8	11494
Cer 18:1;3O/16:0;(2OH)	$C_{34}H_{67}NO_5$	POS	$[M+H]^+$	353.4	570.5088	569.5015	-0.8	-	664.1	14574

Citramalic acid	$C_5H_8O_5$	NEG	$[M+OH]^-$	29.0	165.0407	148.0374	1.3	6.6	-	4452
Citric acid	$C_6H_8O_7$	NEG	$[M-H]^-$	45.1	191.0198	192.0271	0.5	11.1	534.8	5170
Coumaroyl Hexoside	$C_{15}H_{18}O_8$	NEG	$[M-H]^-$	118.6	325.0928	326.1001	-0.2	-	901.6	3884
D-Alanine	$C_3H_7NO_2$	POS	$[M+H]^+$	26.3	90.0550	89.0477	0.2	9.3	-	2026
Diaminopimelic acid	$C_7H_{14}N_2O_4$	POS	$[M+K]^+$	26.3	229.0588	190.0956	1.1	10.3	-	1334
Dihydrokaempferol	$C_{15}H_{12}O_6$	POS	$[M+H]^+$	125.7	289.0703	288.0630	-1.3	-	892.9	10788
Dihydrouracil	$C_4H_6N_2O_2$	NEG	$[M+OH]^-$	20.3	131.0462	114.0429	0.0	-2.2	-	3348
		POS	$[M+K+CH_3CN]^+$	21.4	194.0324	114.0427	-1.0	-1.1	-	1354
			$[M+Na+CH_3CN]^+$	21.9	178.0586	114.0428	-0.8	-0.6	-	3234
Dilinolenin (9c,12c,15c)	$C_{39}H_{64}O_5$	POS	$[M+H]^+$, $[M+H]^+$, $[M+H-H_2O]^+$	337.7	613.4827	612.4756	0.1	-	915.8	22376
Erucamide	$C_{22}H_{43}NO$	POS	$[M+H]^+$, $[M+Na]^+$	338.6	338.3412	337.3339	-1.7	-	908.6	305542
Ethyl-beta-carboline-3-carboxylate	$C_{14}H_{12}N_2O_2$	POS	$[M+H]^+$	176.1	241.0966	240.0893	-2.2	-	679.3	3422
FA 18:1+3O	$C_{18}H_{34}O_5$	NEG	$[M-H]^-$	187.3	329.2325	330.2398	-2.5	-	982.2	29976
Gamma-Caprolactone	$C_6H_{10}O_2$	NEG	$[M+HCOO]^-$	123.5	159.0662	114.0680	-0.3	3.4		3270
Genipin	$C_{11}H_{14}O_5$	POS	$[M+H]^+$	119.4	227.0910	226.0838	-1.7		818.4	8324
Genistein	$C_{15}H_{10}O_5$	NEG	$[M+OH]^-$	153.4	287.0560	270.0527	-0.5	-10.7	-	7720
Gluconic acid	$C_6H_{12}O_7$	NEG	$[M-H]^-$	29.9	195.0512	196.0585	0.9	11.8	600.9	4642
Glucoraphenin	$C_{12}H_{21}NO_{10}S_3$	NEG	$[M-H]^-$	46.9	434.0254	435.0326	-0.3		991.8	56806
Glucosamine	$C_6H_{13}NO_5$	POS	$[M+H]^+$	32.9	180.0864	179.0791	-1.7	16.1	-	1702
Glutamine	$C_5H_{10}N_2O_3$	NEG	$[M-H]^-$, $[M-H-H_2O]^-$	20.8	145.0620	146.0694	0.7	-	895.9	27988
Glutathione	$C_{10}H_{17}N_3O_6S$	NEG	$[M-H]^-$	147.0	306.0759	307.0832	-2.2	-	868.8	7398
		POS	$[M+H]^+$	41.5	308.0903	307.0830	-2.7	-	867.8	4392
Glycerol-myristate	$C_{17}H_{34}O_4$	POS	$[M+H-H_2O]^+$, $[M+H]^+$, $[M+Na]^+$	285.5	285.2418	302.2451	-2.2	-	947.4	43400
Glycylproline	$C_7H_{12}N_2O_3$	NEG	$[M+OH]^-$	29.9	189.0883	172.0850	0.9	7.6	-	1188
Guanidine	CH_5N_3	POS	$[M+K+CH_3CN]^+$	115.6	139.0377	59.0479	-2.9	-	-	3256
Guanosine	$C_{10}H_{13}N_5O_5$	NEG	$[M-H]^-$	52.5	282.0846	283.0919	0.8	-	837.1	2492

		POS	$[M+H]^+$, $[M+Na]^+$, $[M+K]^+$	52.9	284.0986	283.0914	-1.1	-	995.7	8000
Heneicosanoic acid	$C_{21}H_{42}O_2$	POS	$[M+NH_4]^+$	252.4	344.3514	326.3176	-2.6	-	-	5412
Heptadecanoic acid	$C_{17}H_{34}O_2$	POS	$[M+NH_4]^+$	216.9	288.2890	270.2552	-2.5	-	-	8734
Homo-L-arginine	$C_7H_{16}N_4O_2$	POS	$[M+H]^+$	21.9	189.1345	188.1272	-0.8	5.3	-	1802
Indol-3-ylmethyl glucosinolate	$C_{16}H_{20}N_2O_9S_2$	NEG	$[M-H]^-$	113.7	447.0531	448.0604	-1.4		966.7	95776
Indole + 1O, 1carboxy, O-Hex	$C_{15}H_{17}NO_8$	NEG	$[M-H]^-$	97.8	338.0878	339.0950	-1.1	-	740.1	5538
Indole-3-acetyl-L-valine	$C_{15}H_{18}N_2O_3$	POS	$[M+H]^+$	144.5	275.1386	274.1313	-1.5	-	819.6	6914
Indolelactic acid	$C_{11}H_{11}NO_3$	POS	$[M+H+CH_3CN]^+$	145.8	247.1075	205.0736	-1.0	14.7	-	1426
Isobutyric acid	$C_4H_8O_2$	NEG	$[M+HCOO]^-$	99.8	133.0510	88.0528	2.4	-6.6		1166
Kaempferol-3-O-galactoside-7-O-rhamnoside	$C_{27}H_{30}O_{15}$	NEG	$[M-H]^-$	131.0	593.1499	594.1572	-2.1	-	976.6	85706
L-Aspartic acid	$C_4H_7NO_4$	NEG	$[M-H]^-$	29.0	132.0305	133.0378	2.0	11.4	-	1818
		POS	$[M+H]^+$	28.1	134.0445	133.0372	-2.5	10.6	-	1532
Leucylleucine	$C_{12}H_{24}N_2O_3$	POS	$[M+H]^+$	128.4	245.1855	244.1782	-2.0	-	994.0	13150
Leucylproline	$C_{11}H_{20}N_2O_3$	POS	$[M+H]^+$	106.9	229.1544	228.1471	-1.2	-	882.5	7320
Levoglucosan	$C_6H_{10}O_5$	NEG	$[M-H]^-$	0.6	161.0458	162.0531	1.8	-	-	1082
			$[M+HCOO]^-$	29.9	207.0512	162.0530	0.7	-	-	2388
		POS	$[M+Na]^+$	9.1	185.0417	162.0525	-1.9	-	-	4950
			$[M+NH_4]^+$	86.5	180.0870	162.0531	1.7	-	-	1724
L-Histidine	$C_6H_9N_3O_2$	NEG	$[M-H]^-$	20.3	154.0623	155.0696	0.7	4.8	868.3	4820
Linoleic acid	$C_{18}H_{32}O_2$	POS	$[M+NH_4]^+$	235.9	298.2734	280.2395	-2.3	-	-	1314
L-Methyladenosine	$C_{11}H_{15}N_5O_4$	POS	$[M+H]^+$	45.7	282.1194	281.1122	-0.9	-	985.1	4832
L-Norleucine	$C_6H_{13}NO_2$	NEG	$[M-H]^-$	50.7	130.0874	131.0946	0.0	-1.5	-	19408
LPC 18:1	$C_{26}H_{52}NO_7P$	POS	$[M+H]^+$	262.7	522.3544	521.3471	-2.0	-	960.3	22094
LPC 18:2	$C_{26}H_{50}NO_7P$	POS	$[M+H]^+$, $[M+Na]^+$	246.7	520.3385	519.3314	-2.5	-	955.7	50328
LPC 18:3	$C_{26}H_{48}NO_7P$	POS	$[M+H]^+$, $[M+Na]^+$	234.1	518.3231	517.3159	-2.0	-	833.2	180570
LPC 19:1-SN1	$C_{27}H_{54}NO_7P$	POS	$[M+H]^+$, $[M+Na]^+$	278.8	536.3699	535.3629	-2.2	-	883.9	23576
		POS	$[M+H]^+$, $[M+Na]^+$, $[M+H-H_2O]^+$	260.6	454.2918	453.2844	-2.2	-	786.8	102116
LPE 18:1	$C_{23}H_{46}NO_7P$	POS	$[M+H]^+$	261.7	480.3077	479.3004	-1.7	-	804.7	9544

LPE 18:2	$C_{23}H_{44}NO_7P$	POS	$[M+H]^+$, $[M+Na]^+$	245.8	478.2915	477.2842	-2.8	-	788.2	69390
LPE 18:3	$C_{23}H_{42}NO_7P$	POS	$[M+H]^+$, $[M+Na]^+$	232.9	476.2761	475.2689	-2.2	-	656.2	157610
L-Phenylalanine	$C_9H_{11}NO_2$	NEG	$[M-H]^-$	93.3	164.0717	165.0789	-0.3	11.6	780.7	46234
L-Sorbose	$C_6H_{12}O_6$	POS	$[M+NH_4]^+$	33.4	198.0970	180.0632	-1.2	14.8	-	1334
L-Tryptophan	$C_{11}H_{12}N_2O_2$	NEG	$[M-H]^-$, $[2M-H]^-$	109.5	203.0826	204.0899	0.1	12.9	779.3	124094
		POS	$[M+Na]^+$	108.3	227.0790	204.0898	-0.3	11.7	-	5314
Valine	$C_5H_{11}NO_2$	NEG	$[M-H]^-$	35.8	116.0719	117.0792	1.8	13.4	-	1732
		POS	$[M+Na]^+$	38.7	140.0680	117.0788	-1.5	16.3	-	1096
Malic acid	$C_4H_6O_5$	NEG	$[M-H]^-$	141.0	133.0142	134.0214	-0.7	-	709.7	21194
		POS	$[M+Na]^+$	34.8	157.0104	134.0212	-2.3	13.1		2618
m-Coumaric acid	$C_9H_8O_3$	NEG	$[M-H]^-$	138.0	163.0399	164.0472	-1.0	6.7	972.6	49736
		POS	$[M+NH_4]^+$	137.1	182.0808	164.0470	-2.1	5.8	-	1214
Methyl propenyl ketone	C_5H_8O	NEG	$[M+HCOO]^-$	119.6	129.0558	84.0576	0.3	-5.7	-	2898
Methylsuccinic acid	$C_5H_8O_4$	POS	$[M+Na]^+$	8.7	155.0311	132.0419	-2.4	-	-	1024
Monolaurin	$C_{15}H_{30}O_4$	POS	$[M+H-H_2O]^+$, $[M+Na]^+$, $[M+H]^+$	257.8	257.2108	274.2137	-1.3	-	701.8	4654
N-Acetyldihydrosphingosine	$C_{20}H_{41}NO_3$	POS	$[M+H]^+$	244.4	344.3152	343.3079	-2.2	-	601.0	7808
N-Acetylglutamic acid	$C_7H_{11}NO_5$	NEG	$[M-H]^-$	32.9	188.0567	189.0640	1.4	10.5	-	1330
		POS	$[M+H]^+$	30.2	190.0708	189.0636	-0.8	7.8	-	1474
N-Acetyl-L-alanine	$C_5H_9NO_3$	NEG	$[M-H]^-$	48.9	130.0510	131.0582	-0.1	3.2	-	1236
		POS	$[M+H+CH_3CN]^+$	44.9	173.0916	131.0577	-2.9	-0.9	-	2378
NAOrn 15:0/16:0	$C_{36}H_{70}N_2O_5$	POS	$[M+H]^+$, $[M+Na]^+$	307.3	611.5357	610.5281	0.0	-	824.3	3850
NAOrn 16:0/16:0	$C_{37}H_{72}N_2O_5$	POS	$[M+H]^+$	312.2	625.5509	624.5436	-0.8	-	919.9	7314
NAOrn 16:0/18:1	$C_{39}H_{74}N_2O_5$	POS	$[M+H]^+$, $[M+Na]^+$	312.2	651.5676	650.5603	0.9	-	895.0	35392
NAOrn 16:1/16:0	$C_{37}H_{70}N_2O_5$	POS	$[M+H]^+$, $[M+Na]^+$	306.2	623.5355	622.5287	-0.4	-	898.0	6520
NAOrn 16:1/18:1	$C_{39}H_{72}N_2O_5$	POS	$[M+H]^+$	306.5	649.5518	648.5446	0.7	-	868.0	6424
NAOrn 18:1/18:1	$C_{41}H_{76}N_2O_5$	POS	$[M+H]^+$, $[M+Na]^+$	312.7	677.5839	676.5770	1.8	-	887.4	14648
	$C_{26}H_{28}O_{14}$	NEG	$[M-H]^-$	131.0	563.1396	564.1469	-1.8	-	748.7	5440

5-Hydroxy-2-(4-hydroxyphenyl)-7-(3,4,5-trihydroxy-6-methyloxan-2-yl)oxy-3-(3,4,5-trihydroxyoxan-2-yl)oxychromen-4-one		POS	$[M+H]^+$, $[M+Na]^+$	131.7	565.1547	564.1470	-0.9	-	993.6	8332
5,7-Dihydroxy-2-(4-hydroxyphenyl)-3-[(2S,3R,4R,5R,6S)-3,4,5-trihydroxy-6-methyloxan-2-yl]oxychromen-4-one	$C_{21}H_{20}O_{10}$	POS	$[M+H]^+$, $[M+NH_4]^+$, $[M+Na]^+$	163.2	433.1121	432.1049	-1.9	-	966.5	3974
(2R,3S)-2-(4-Hydroxyphenyl)-3,4-dihydro-2H-chromene-3,5,7-triol	$C_{15}H_{14}O_5$	POS	$[M+H]^+$	120.8	275.0917	274.0844	1.0	-	874.2	3716
3-Oxo-3-[[(2R,3S,4S,5R,6S)-3,4,5-trihydroxy-6-[5-hydroxy-3-(4-hydroxyphenyl)-4-oxochromen-7-yl]oxyoxan-2-yl]methoxy]propanoic acid	$C_{24}H_{22}O_{13}$	POS	$[M+H]^+$	140.2	519.1126	518.1053	-1.4	-	918.4	3062
8-{(1S,5R)-4-Oxo-5-[(2Z)-2-penten-1-yl]-2-cyclopenten-1-yl}octanoic acid	$C_{18}H_{28}O_3$	POS	$[M+H-H_2O]^+$, $[M+H]^+$	180.0	275.2003	292.2036	-0.9	-	732.1	33206
Nonadecanoic acid	$C_{19}H_{38}O_2$	POS	$[M+NH_4]^+$	237.1	316.3202	298.2864	-2.5	-	-	3288
			$[M+Na+CH_3CN]^+$	313.6	362.3024	298.2867	-1.4	-	-	1224
Octadecanol	$C_{18}H_{38}O$	POS	$[M+H+CH_3CN]^+$	339.7	312.3255	270.2917	-1.8	-	-	1960
Octanal	$C_8H_{16}O$	NEG	$[M+HCOO]^-$	185.4	173.1182	128.1200	-0.4	-	-	1006
Oleamide	$C_{18}H_{35}NO$	POS	$[M+H]^+$, $[M+Na]^+$	305.6	282.2786	281.2713	-1.8	-	961.4	174782
Oxalic acid	$C_2H_2O_4$	NEG	$[M-H]^-$	18.3	88.9882	89.9955	2.4	-	-	1330
Oxoglutaric acid	$C_5H_6O_5$	NEG	$[M-H]^-$	37.6	145.0144	146.0217	1.2	13.5	-	2330
Palmitamide	$C_{16}H_{33}NO$	POS	$[M+H]^+$	301.9	256.2632	255.2559	-1.3	-	945.0	25596
Palmitic Acid	$C_{16}H_{32}O_2$	POS	$[M+H]^+$	309.6	257.2471	256.2398	-1.8	-	789.2	5336
Pantothenic acid	$C_9H_{17}NO_5$	POS	$[M+H+CH_3CN]^+$	55.8	261.1440	219.1102	-1.9	-	-	1702
PC O-16:1	$C_{24}H_{48}NO_7P$	POS	$[M+H]^+$, $[M+Na]^+$	238.5	494.3230	493.3159	-2.3	-	966.0	5280
PC(16:0/0:0)	$C_{24}H_{50}NO_7P$	POS	$[M+H]^+$, $[M+K]^+$, $[M+Na]^+$	261.7	496.3387	495.3315	-2.1	-	904.2	186532
PE O-13:0_5:0	$C_{23}H_{48}NO_7P$	POS	$[M+H]^+$, $[M+Na]^+$	287.3	482.3232	481.3154	-1.9	-	945.2	17408

PE O-13:1_3:0	$C_{21}H_{42}NO_7P$	POS	$[M+H]^+$, $[M+Na]^+$	237.6	452.2763	451.2692	-1.9	-	942.3	9406
PE O-16:1_3:0	$C_{24}H_{48}NO_7P$	POS	$[M+H]^+$, $[M+Na]^+$	278.1	494.3231	493.3156	-2.0	-	913.7	12352
Phenylacetic acid	$C_8H_8O_2$	NEG	$[M-H]^-$	128.2	135.0450	136.0523	-1.0	-14.2	-	1466
Phenylalanylisoleucine	$C_{15}H_{22}N_2O_3$	POS	$[M+H]^+$	136.1	279.1697	278.1625	-2.1	-	871.4	8064
Pheophorbide A	$C_{35}H_{36}N_4O_5$	POS	$[M+H]^+$, $[M+Na]^+$	310.6	593.2753	592.2682	-1.0	-	946.2	115272
Phloridzin	$C_{21}H_{24}O_{10}$	NEG	$[M-H]^-$	121.5	435.1295	436.1368	-0.3	-	943.2	2928
Phosphoric acid	H_3O_4P	POS	$[M+H]^+$	1.0	98.9840	97.9767	-2.1	-17.2	-	1308
Phosphorylcholine	$C_5H_{15}NO_4P$	POS	$[M+NH_4]^+$	135.6	202.1075	184.0736	-1.2	-	-	1722
Phytosphingosine	$C_{18}H_{39}NO_3$	POS	$[M+H]^+$	228.8	318.2994	317.2922	-2.6	-	884.8	11744
Pipecolic acid	$C_6H_{11}NO_2$	POS	$[M+Na]^+$	10.0	152.0679	129.0787	-2.1	-12.4	-	1042
			$[M+NH_4]^+$	19.3	147.1125	129.0786	-2.3	-3.0	-	1866
Pipericine	$C_{22}H_{41}NO$	POS	$[M+H]^+$	325.0	336.3256	335.3184	-1.4		694.6	6212
Pregnenolone sulfate	$C_{21}H_{32}O_5S$	POS	$[M+NH_4]^+$	180.6	414.2297	396.1958	-2.9	-6.6	-	1310
Propanal	C_3H_6O	NEG	$[M+HCOO]^-$	10.9	103.0404	58.0422	2.8	-9.3	-	1202
		POS	$[M+Na]^+$	9.7	81.0313	58.0421	2.8	-10.5	-	14794
Pyroglutamic acid	$C_5H_7NO_3$	NEG	$[M+OH]^-$	22.2	146.0461	129.0428	1.7	-0.3	-	3406
		POS	$[M+H]^+$, [M+Na]+, $[M+K]^+$, $[M+H-CO_2]^+$	22.4	130.0495	129.0431	-2.6	-0.2	903.7	59616
Pyromucic acid	$C_5H_4O_3$	NEG	[M+OH]-	46.9	129.0195	112.0162	1.3	-	-	2400
Pyrrolidonecarboxylic acid	$C_5H_7NO_3$	POS	[M+NH4]+	376.4	147.0760	129.0422	-2.8	-	-	1092
Pyruvic acid	$C_3H_4O_3$	NEG	$[2M-H]^-$, $[M-H]^-$	32.6	175.0250	88.0162	1.0	-	731.5	18724
Rhamnose	$C_6H_{12}O_5$	NEG	$[M+HCOO]^-$	31.0	209.0667	164.0685	0.3	11.1	-	1968
Salicin	$C_{13}H_{18}O_7$	NEG	$[M-H]^-$	155.3	285.0971	286.1044	-3.0		-	1520
Sebacic acid	$C_{10}H_{18}O_4$	NEG	$[M-H]^-$	166.4	201.1133	202.1206	0.2	14.6	-	1302
Shikimic acid	$C_7H_{10}O_5$	NEG	$[M+OH]^-$	29.0	191.0565	174.0532	2.0	6.8	-	1158
Sphinganine	$C_{18}H_{39}NO_2$	POS	$[M+H]^+$	240.4	302.3048	301.2975	-1.9	17.1	-	1532
Sphingosine	$C_{18}H_{37}NO_2$	POS	$[M+H]^+$	231.4	300.2889	299.2817	-2.6	11.5	-	2440
Stearic acid	$C_{18}H_{36}O_2$	POS	$[M+H-H_2O]^+$, $[M+H]^+$	327.5	267.2675	284.2712	-2.8	-2.6	-	3916
Succinic acid	$C_4H_6O_4$	NEG	$[M-H]^-$, $[M-H-H2O]^-$	48.9	117.0194	118.0267	0.1	10.6	773.8	49584

		POS	$[M+Na]^+$	40.4	141.0162	118.0270	2.9	2.0	-	1538
Succinic acid semialdehyde	$C_4H_6O_3$	NEG	$[M-H]^-$	32.9	101.0245	102.0318	0.7	-	-	1214
Sucrose	$C_{12}H_{22}O_{11}$	POS	$[M+NH_4]^+$	34.8	360.1497	342.1159	-1.0	17.1	651.1	3534
Sulfojasmonate	$C_{12}H_{18}O_7S$	NEG	$[M-H]^-$	124.4	305.0694	306.0767	-2.2	-	671.3	9038
Tetracosanoic acid	$C_{24}H_{48}O_2$	POS	$[M+NH_4]^+$	278.1	386.3983	368.3645	-2.4	-	-	1094
Trehalose	$C_{12}H_{22}O_{11}$	NEG	$[M+HCOO]^-$, $[M-H]^-$	35.3	387.1145	342.1163	0.2	14.6	-	19064
Tryptamine	$C_{10}H_{12}N_2$	POS	$[M+NH_4]^+$	110.9	178.1334	160.0996	-2.5	7.5	-	1642
Tryptophanol	$C_{10}H_{11}NO$	NEG	[M+HCOO]-	138.0	206.0821	161.0839	-0.6	-5.7	-	3110
Umbelliferone	$C_9H_6O_3$	NEG	$[M+OH]^-$	128.2	179.0346	162.0313	-2.4	-	-	3956
		POS	$[M+H]^+$	149.4	163.0386	162.0313	-2.5	-	-	1030
Ureidopropionic acid	$C_4H_8N_2O_3$	POS	$[M+Na]^+$	21.9	155.0424	132.0532	-1.8	-0.6	-	2686
			$[M+K]^+$	22.4	171.0165	132.0533	-1.0	-0.1	-	1150
			$[M+H]^+$	29.0	133.0604	132.0531	-3.0	6.5	-	5590
Uridine diphosphategalactose	$C_{15}H_{24}N_2O_{17}P_2$	NEG	$[M-H]^-$	32.9	565.0477	566.0550	-0.1	14.3	-	3350
Vanillylmandelic acid	$C_9H_{10}O_5$	NEG	$[M+OH]^-$	140.0	215.0563	198.0530	0.8	-	-	1164
Xanthurenic acid	$C_{10}H_7NO_4$	NEG	$[M-H]^-$	109.0	204.0303	205.0376	0.2	13.8	-	3320
		POS	$[M+H]^+$	108.3	206.0447	205.0374	-0.6	13.1	-	2430

[a]Experimental monoisotopic mass of ion; [b]Theoretical monoisotopic mass of compound; *m/z*: mass-to-charge ratio; NEG: ionization in negative mode; POS: ionization in positive mode; RT: retention time; $[M+OH]^-$ ions are in fact $[M+H_2O-H]^-$ ions.

S7. Results of optimization of ablation resolution

Optimization was carried out by ablating a 6x6x1 voxel raster on the surface of the frozen agar gel (with titanium dioxide). Laser shooting time 1 s (20 shots). Please, note that the agar gel was locally melted and deformed (for example, for a resolution of 100 µm). The results of the ablation were analyzed with the use of the built-in distance sensor and are provided below as images of 3D models. The smallest deviation from the average depth level of the bottom of ablated area was considered the best result.

Resolution 100 µm: average depth 590 µm, deviation from average depth level: 60 µm

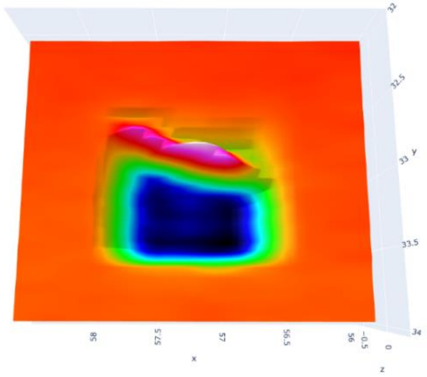

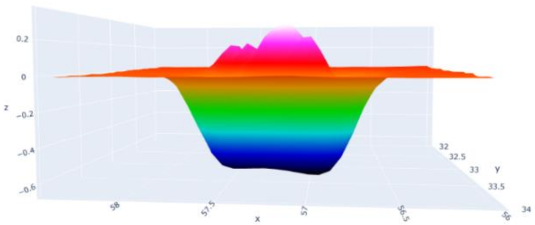

Resolution 120 µm: average depth 470 µm, deviation from average depth level: 30 µm

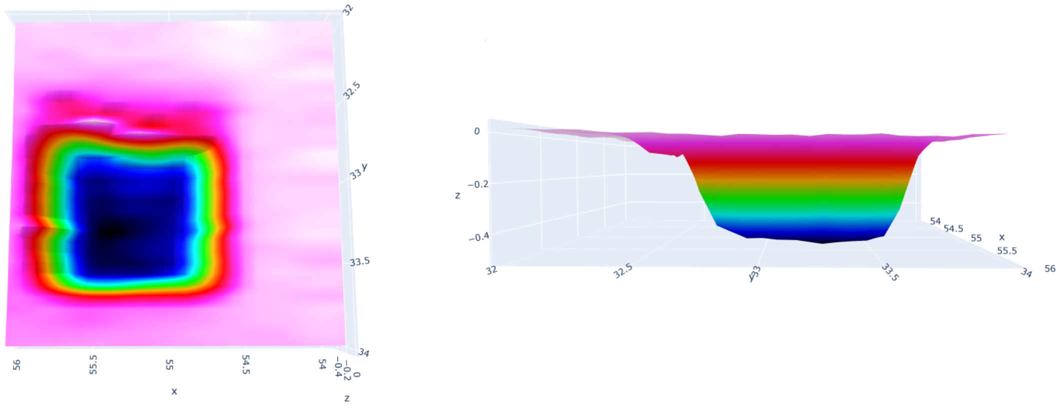

Resolution 140 µm: average depth 360 µm, deviation from average depth level: 20 µm

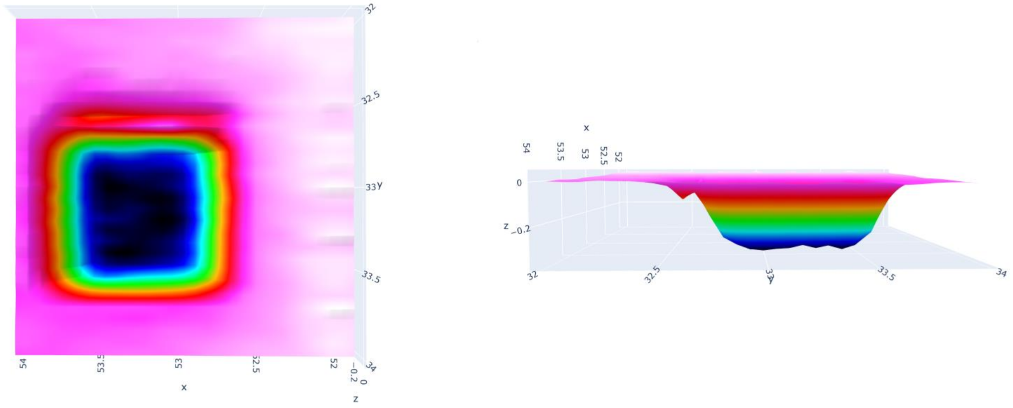

Resolution 150 µm: average depth 310 µm, deviation from average depth level: 60 µm

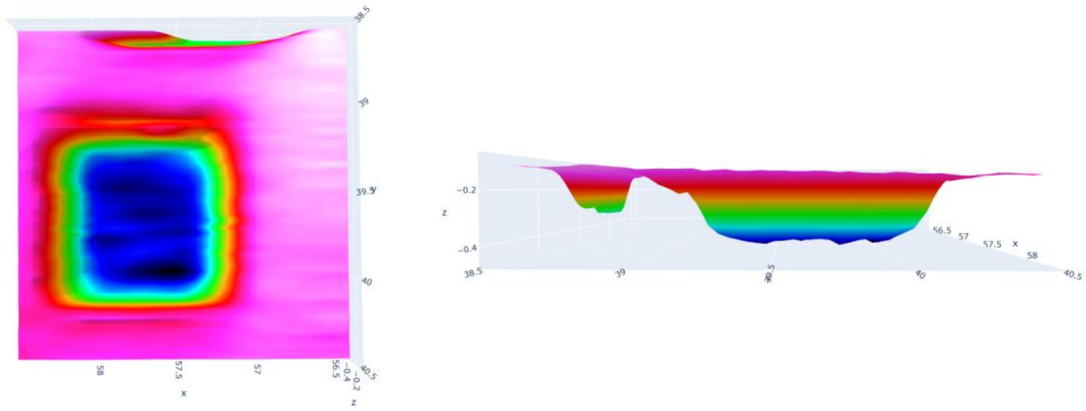

Resolution 160 µm: average depth 250 µm, deviation from average depth level: 40 µm

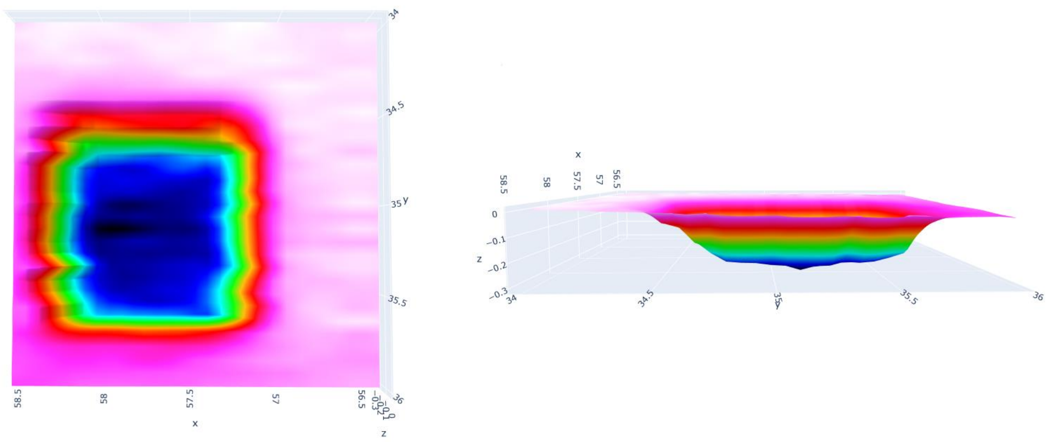

Resolution 180 µm: average depth 200 µm, deviation from average depth level: 30 µm

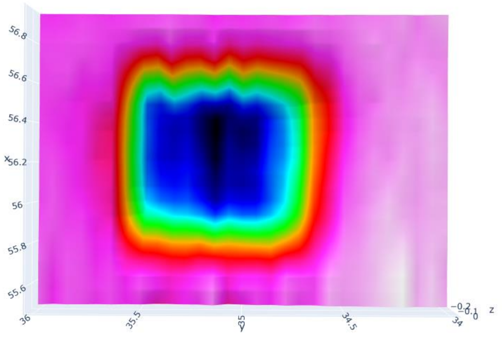

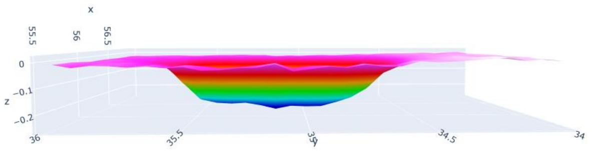

S8. LARAPPI/CI 2D and 3D MSI control and analysis software

We have developed two programs locally to enhance our MSI research. Both applications are built using .NET 8 and Windows Presentation Foundation (WPF), featuring modern user interfaces (UIs) and a configuration layer with a decoupled logic system that utilizes an in-process messaging system.

The control software enables manual and automated MSI experiments using a sophisticated commanding system and queues. It defines multiple types to control various external devices, including (i) movement of the sample along the XYZ axes with controlled speed and acceleration, laser shooting, and relays for nitrogen pressurizing, chamber lighting, and cooling systems. Each command offers numerous customizable parameters through the user interface, such as displaying a raster of the ablation points in the selected sample region. This ensures that all parameters are correct before starting the experiment (e.g., pixel overlap or movement velocity). The software registers all relevant data in real time, such as timestamps (with 100 µs) and XYZ coordinates (better than 3 µs accuracy), and allows visual inspection of samples through an installed camera. It also functions as a profilometer, generating 2D and 3D charts based on XYZ and distance sensor data. The built-in calibration methods ensure that all devices operate at the same coordinates, eliminating errors.

Visualization software is used to open MSI scans using Bruker.d files alongside control-software-generated ones. Supports opening a single experiment for 2D or multiple experiments for 3D. The software directly reads Bruker data for each layer, merging them into a custom, search-friendly, and optimized structure upon first load, allowing for near-instant data queries. Additional optimization techniques are applied. Before the experiments, synchronization of all layers to ensure correct centering of the MS chromatogram data by setting time offsets. The software supports multiple types of acquisition (MS and BBCID in particular). It is possible to work with each layer of a 3D scan individually and interact with the parent 3D structure. The 2D visualization features a detailed heatmap, while the 3D visualization consists of a custom-built stage in a 3D graphing

library with volumetric plots where the peak ion intensity is represented by opacity. Visualization software has recently been updated to support additional volumetric rendering using the Unity3D engine (version 2022.3), significantly enhancing the presentation of 3D ion data. This update provides greater rendering capabilities, allowing for improved resolution, lighting, and overall data visualization.

S9. Materials and methods

Participant

The study protocol was approved by the Bioethics Committee of the University of Rzeszow (Poland), and the research was carried out according to relevant guidelines and regulations. Specimens and clinical data from patients involved in the study were collected with informed consent. All laboratory tests for the cancer patient (complete blood count, kidney function tests, CRP, urine analysis, bleeding profile) were within normal limits. A whole tumor with a small fragment of adjacent healthy tissue was resected (cancer and control tissue, respectively). Control tissue was recognized as normal on pathological analysis.

Preparation of agar gel with titanium dioxide

An amount of 1440 mg of agar (BLT sp. z o. o., Poland) was placed in a beaker with 32 mL of deionized water. Titanium dioxide nanopowder (1g, Aldrich) was added and mixed until a uniformly opaque white color was reached. The solution was heated on a hot plate until the agar was completely dissolved. The warm gel was poured into a glass Petri dish and left to solidify at 4 ° C.

Preparation of agar gel with test compounds

An amount of 1480 mg of agar (BLT sp. z o. o., Poland) was placed in a beaker with 32 mL of deionized water. The solution was heated on a hot plate until the agar was completely dissolved. To the hot agar gel, 3.2 mg of each test compound (ribose, histidine, thymidine, uracil) were added and mixed. The warm gel was poured into a glass Petri dish and left to solidify at 4 ° C.

LARAPPI/CI optimization: Selection of ionization mode

Agar gel with dissolved test compounds of 3 x 3 cm size was placed on the sample table in the pressure chamber of the LARAPPI/CI system. After freezing of gel to -18°C temperature (temperature measured of sample table) 7 x 7 voxels rasters were programmed (pixel-to pixel delay 3 s, line-to line delay 5 s) and laser ablation (20 laser pulses, 20 Hz) executed. The mass spectrometer was working in MS^1 mode with 5 Hz frequency of spectra collection with *m/z* range 50-1000.

LC-MS sample preparation

Metabolomic profiling was performed on a red radish sample from tissue adjacent to the one studied by 3D MSI. Approximately 200 mg of the sample was weighed and cut into small pieces. To the sample pieces, 150 µL of distilled water and 900 µL of acetone (Sigma, Aldrich, LC-MS grade) were added. At the same time, 3 stainless beads were added to this mixture, and the sample was homogenized 3 times for one minute with BeadBug 6 (Benchmark Scientific) at maximum speed. Subsequently, the sample was incubated at temp. 4°C overnight. The following day, the sample was centrifuged (mySPIN™ 12 Mini Centrifuge, Thermo Fisher Scientific) at conditions: temp. 4°C, 14,000×g for 5 minutes. The supernatant was transferred, and the sample was left in a speed vac-type apparatus (2E-3 mbar vacuum, 1300 rpm) overnight. The next day, the dried pellet was dissolved in methanol (800 µL) To facilitate dissolution, the sample was sonicated and then centrifuged for 5 minutes at 14,000×g, and the resulting supernatant was transferred to a standard HPLC vial.

LC-MS Metabolomic Analysis

Mass spectrometry-liquid chromatography analyzes were performed on a Bruker Elute UHPLC system operated by Hystar 3.3 software and an ultrahigh-resolution (60000+) mass spectrometer Bruker Impact II (Bruker Daltonik GmbH) ESI QTOF-MS equipped with Data Analysis 4.2 (Bruker Daltonik GmbH), TASQ (2022b) and Metaboscape (2022b). The ion source used was a Bruker VIP-HESI with optimized flows and temperatures. The column used for AutoMSMS measurements was Waters Acquity UPLC BEH C18 1.7 µm particles of 2.1 x50 mm dimensions. For AutoMSMS measurements flows and percentages were: 0 and 0.56 min 99% A, 4.72 min - 1% A, 5.56 min - 1% A, 5.60, 6.34, and 9.45 min – 99% A, all flows at 450 $\mu Lmin^{-1}$. The column was held at 40°C.

The column exit was connected to the VIP HESI ion source. Internal calibration on 10 mM sodium formate (water: isopropanol 1:1 v/v) ions was performed automatically in Metaboscape with the use of a syringe pump at an infusion flow rate of 0.12 mL h^{-1}, using a high precision calibration (HPC) mode. The autoMSMS method was used with *m/z* range 50-1500; CID (Collision-Induced Dissociation) was used with the following settings: absolute area threshold: 5000 counts; active exclusion 2 spectra; release after 0.3 min, isolation mass: for *m/z* = 100, width was 4, for 300 width was 5, for 500 was 6 and for 1000 was 8): 15, 10, 5 eV; collision energy value was 30 eV.

The untargeted annotations were performed in Metaboscape (ver. 2022b) with a criterion of mass deviation (Δ*m/z*) under 2 ppm and mSigma value under 30 as the maximum acceptable deviation of the mass of the compound and the isotopic pattern respectively. All the molecular formulas were obtained using the Smart Formula tool and the C, H, N, O, P, S, Cl, Br, I, and F elements. MSMS spectra were automatically matched with MSMS libraries: Bruker HMDB 2.0 library, MassBank of North America (MoNA) library [68], and NIST ver. 2020 MSMS library [69]. For compounds annotated in Metaboscape 2D and 3D ion images were generated, and some of them are shown in this work.

Parameters for 3D MSI experiments

The Bruker Impact II QToF mass spectrometer (resolution 60000+) was used in negative ion mode in MS or MS+bbCID (broadband collision-induced dissociation) modes. The settings of the ion source were as follows: APCI nebulizer, end plate offset 600V, capillary 1000V, corona 6000 nA, nebulizer 3.5 bar, dry gas 0.2 L/min, dry temperature 250°C, probe gas temperature 350°C, probe gas 4 L/min, exhaust turned on. MS-only experiments were performed with the following settings: scan range *m/z* 47-1300, MS frequency 7 Hz. The MS + bbCID experiments also used bbCID settings: collision energy MS: 7 eV, bbCID: 25 eV, acquisition time factor 1x+1x, frequency 5Hz. The rest of the settings were the default for the metabolomic method provided by Bruker.

3D MSI experiments for kiwi fruit and red radish tissues were carried out in negative ion MS mode, while human tissue experiment was carried out in negative ion MS+bbCID measurement mode. All 3D experiments were performed with 10 laser pulses per voxel. For kiwi fruit tissue, the upper ablation level was made in 140 µm resolution in 35 x 35 (X x Y) voxel arrangement, second (lower) level – 34 x 34, third – 33 x 33, fourth – 32 x 32, fifth – 31 x 31, sixth – 30 x 30 with total depth after all six ablation steps – 1.43 mm (average 238 µm per level). For red radish tissue, the resolution and voxel arrangement were identical to that for kiwi tissue with total depth of 1.67 mm (average 278 µm per level). Human kidney tissue was measured as follows: first layer – 35 x 35 voxels, second – 34 x 34, third – 33, fourth – 32 x 32, total depth – 0.95 mm (avg. 238 µm per level). An example of voxel arrangement for 6 levels starting at 35 x 35 is presented in Figure 2G. All objects were of 5 mm thickness and sizes 25 x 9 (kidney tissue), red radish – 15 x 25 and 25 x 20 mm in case of kiwi. The mentioned tissues were cut with a blade and placed on a stainless steel plate, then on an ablation table inside the chamber, and frozen. The software used to perform 2D and 3D MSI experiments, as well as analysis of the data, is described in Supporting Information S8.

Table S1. Comparison of the efficiency of ionization methods performed by ablation of agar gel containing test compounds and mass spectrometric measurements.

Ionization method	Compound	Ion formula[a]	S/N[b]	$\|m/z_{calc.}-m/z_{exp.}\|$
APCI negative mode	ribose	$[M-H]^-$	42.6	0.0037
	histidine	$[M-H]^-$	10	0.0041
	thymidine	$[M-H]^-$	26.4	0.0063
	uracil	$[M-H]^-$	14.5	0.0028
APCI/APPI negative mode	ribose	$[M-H]^-$	225.6	0.0038
	histidine	$[M-H]^-$	14.3	0.0042
	thymidine	$[M-H]^-$	50.6	0.0063
	uracil	$[M-H]^-$	113.9	0.0028
APCI positive mode	ribose	$[M+H]^+$	1.4	0.0067
	histidine	ND	ND	-
	thymidine	ND	ND	-
	uracil	ND	ND	-
APCI/APPI positive mode	ribose	$[M+Na]^+$	2.7	0.0071
	histidine	$[M+H]^+$	1.8	0.0078
	thymidine	$[M+H]^+$	4.0	0.0143
	uracil	$[M+H]^+$	3.7	0.0061
ESI/APPI negative mode	ribose	$[M-H]^-$	7.8	0.0502
	histidine	$[M-H]^-$	11.8	0.0012
	thymidine	$[M-H]^-$	38.5	0.0019
	uracil	$[M-H]^-$	23.4	0.0009
ESI/APPI positive mode	ribose	ND	ND	-
	histidine	$[M+H]^+$	6.0	0.0089
	thymidine	$[M+H]^+$	9.0	0.0146
	uracil	ND	ND	-

[a]The highest intensity ion detected for a given compound; [b]Signal-to-noise ratio for the mass spectrum recorded during laser ablation of the test

agar gel; ND – not detected; $m/z_{calc.}$ – calculated m/z value; $m/z_{exp.}$ – experimental *m/z* value.
